# Frag4Lead: growing crystallographic fragment hits by catalog using fragment-guided template docking

**DOI:** 10.1107/S2059798321008196

**Published:** 2021-08-23

**Authors:** Alexander Metz, Jan Wollenhaupt, Steffen Glöckner, Niki Messini, Simon Huber, Tatjana Barthel, Ahmed Merabet, Hans-Dieter Gerber, Andreas Heine, Gerhard Klebe, Manfred S. Weiss

**Affiliations:** a Department of Pharmaceutical Chemistry, Philipps-University Marburg, Marbacher Weg 6, D-35032 Marburg, Germany; b Macromolecular Crystallography, Helmholtz-Zentrum Berlin, Albert-Einstein-Straße 15, D-12489 Berlin, Germany

**Keywords:** crystallographic fragment screening, fragment-based lead discovery, template docking, structure-based drug design, pose validation

## Abstract

In recent years, crystallographic fragment screening has matured into an almost routine experiment at several modern synchrotron sites. The hits of the screening experiment, *i.e.* small molecules or fragments binding to the target protein, are revealed along with their 3D structural information. Therefore, they can serve as useful starting points for further structure-based hit-to-lead development. However, the progression of fragment hits to tool compounds or even leads is often hampered by a lack of chemical feasibility. As an attractive alternative, compound analogs that embed the fragment hit structurally may be obtained from commercial catalogs. Here, a workflow is reported based on filtering and assessing such potential follow-up compounds by template docking. This means that the crystallographic binding pose was integrated into the docking calculations as a central starting parameter. Subsequently, the candidates are scored on their interactions within the binding pocket. In an initial proof-of-concept study using five starting fragments known to bind to the aspartic protease endothiapepsin, 28 follow-up compounds were selected using the designed workflow and their binding was assessed by crystallography. Ten of these compounds bound to the active site and five of them showed significantly increased affinity in isothermal titration calorimetry of up to single-digit micromolar affinity. Taken together, this strategy is capable of efficiently evolving the initial fragment hits without major synthesis efforts and with full control by X-ray crystallography.

## Abstract 2

A workflow to expand crystallographic fragment hits to higher affinity compounds using readily available analogs is described and successfully applied.

## 1. Introduction   

In a drug-discovery project, the hits obtained by fragment screening are typically smaller than the lead-like molecules obtained from a high-throughput screening (HTS) campaign. Nonetheless, fragments constitute excellent starting points for lead discovery as they usually explore the hotspots of binding, where a large part of the binding affinity can be obtained. It is clear, however, that owing to their small size and their weak binding affinity, fragments need to be improved with respect to affinity and specificity. Also, due to their rather small number of interactions with the protein surface, fragments are often promiscuous binders. Subsequent optimization can usually be achieved more efficiently compared with HTS hits, as fragments leave sufficient space and options for exit vectors to expand and improve binding upon optimization. In the past, numerous fragment-screening methods have been established to detect such starting points for follow-up lead discovery (Erlanson *et al.*, 2016 ▸). The increasing popularity of these approaches is reflected by the growing number of reported fragment-to-lead campaigns (Mortenson *et al.*, 2019 ▸) and, consequently, a large number of candidates have entered clinical trials (Erlanson *et al.*, 2016 ▸). Meanwhile, four approved drugs developed by fragment-based lead discovery (FBLD) have been launched to market. To efficiently accomplish such hit-to-lead-to-drug developments, the support of X-ray crystal structure analysis is essential, as validated binding modes allow the immediate application of structure-based design concepts to the subsequent optimization process (Murray & Rees, 2016 ▸; Schmidt & Rademann, 2009 ▸). Therefore, crystallographic fragment screening (CFS), if applicable, has major advantages over alternatives such as HTS based on biochemical or biophysical assays, which are mostly in need of target-binding validation and binding-mode characterization before moving forward into efficient structure-based optimization (Schiebel, Krimmer *et al.*, 2016 ▸; Schiebel, Radeva *et al.*, 2016 ▸). Recent improvements in instrumentation at several synchrotron beamline facilities, as well as in automated data-handling procedures, have greatly improved the capabilities of CFS. Consequently, CFS can be performed with relatively little effort, also enabling access for academic groups experienced in crystallographic methods (Schiebel, Krimmer *et al.*, 2016 ▸; Lamoree & Hubbard, 2017 ▸; Wollenhaupt *et al.*, 2021 ▸; Lima *et al.*, 2020 ▸; Krojer *et al.*, 2017 ▸). Based on screening collections of some 100–1000 compounds, hit rates of 0.5–10% have been achieved with CFS (Hartshorn *et al.*, 2005 ▸). More recently, however, improved libraries have elevated the hit rates to 15–30% (Schiebel, Krimmer *et al.*, 2016 ▸; Wollenhaupt *et al.*, 2020 ▸), or even above 40% for very low molecular mass fragments (O’Reilly *et al.*, 2019 ▸). As a matter of fact, these developments have shifted the initial bottleneck of finding starting points from hit detection per se towards the subsequent progression of the fragment hits into ligands with improved affinity and selectivity.

Fragment-hit optimization towards higher affinity compounds usually involves elaborate chemical synthesis with follow-up medicinal chemistry at a relatively early stage (Murray & Rees, 2016 ▸). To facilitate this process, fragment libraries can be designed and assembled in such a way that discovered hits can be easily expanded to provide entry points into larger chemical spaces (Cox *et al.*, 2016 ▸; Keserű *et al.*, 2016 ▸). In the case where a strong and experienced medicinal chemistry synthesis group is not within reach, the further progress of drug-development projects, particularly in academic settings, is easily hindered or sometimes even completely stalled, mostly in the initial phase of a lead-finding process (Murray & Rees, 2016 ▸; Chevillard & Kolb, 2015 ▸).

As the first step of a fragment-to-lead campaign, the initial fragment hits require some validation in order to ensure that reasonably close analogs of the identified hits bind in a similar fashion. If no such analogs can be identified, the fragment may be hard to optimize or may present a case with binding modes that easily swap upon minor chemical modification. The determination of the binding poses of structurally closely related fragments provides confidence in the reliability and relevance of an observed fragment hit and its pose, and often allows the development of an initial crude structure–activity relationship (SAR). This can be achieved by simply exploring readily available analogs in an ‘SAR-by-catalog’ approach (Erlanson *et al.*, 2019 ▸; Schulz *et al.*, 2011 ▸). In fortunate cases, suitable analogs can be further evaluated by structure-based computational methods, in particular by molecular docking (Yuriev & Ramsland, 2013 ▸). However, the identification of a promising scaffold with the correct binding pose among a large variety of possibilities via docking still remains a challenging problem, especially for molecules as small as fragments that form only a few interactions and can easily alter their binding poses upon modulation of their substitution patterns (Lamoree & Hubbard, 2017 ▸; Oebbeke *et al.*, 2021 ▸).

To efficiently exploit hits from CFS, methods are needed to either suggest easily accessible structural and chemical analogs of a given hit to validate its binding pose or to retrieve commercially available larger compounds embedding the initial hit. Such analogs can be retrieved by web interfaces that are often provided by the vendors or vendor aggregators themselves. Among the most used aggregators are MolPort, Chemspace, eMolecules, Mcule, Enamine and LabNetwork. These catalogs are now also conveniently interfaced by overarching tools such as *Manifold* (https://postera.ai/manifold/), which is free for academic use. Other approaches to visualize the search for effective SARs have also been reported recently (Hall *et al.*, 2017 ▸). Nonetheless, efficient CFS hit exploitation requires strategies to prioritize the list of suitable follow-up candidates from the possibly vast number of commercially available analogs. This prioritization of potential follow-up compounds is best supported by computational tools and ideally exploits the crystallographic knowledge of the bound fragment as a template to guide the next design steps by the virtual screening of candidates (de Souza Neto *et al.*, 2020 ▸). In our approach, the additional chemical groups of a putative follow-up candidate are tethered to the original fragment hit in its bound state. In this regard, the information obtained by CFS is combined with a computational growth strategy.

In order to demonstrate the concept of our developments, the aspartyl protease endothiapepsin (EP), an enzyme frequently used to develop principles and novel strategies in inhibitor design, was used as the target protein. Five hits from a previous CFS campaign (Radeva, Krimmer *et al.*, 2016 ▸; Radeva, Schiebel *et al.*, 2016 ▸; Schiebel, Krimmer *et al.*, 2016 ▸) were used to emulate a real-case scenario with only a few and potentially non-optimal fragment hits available. This means that these hits do not reflect a prioritized selection of the 41 binders that address the catalytic dyad of EP, but instead contain direct and indirect dyad binders and span a wide range of affinities (100 µ*M* to 8.8 m*M*). Additionally, only a limited number of commercially available follow-up candidates were tested. From the selected 28 follow-up compounds, ten binders could be identified by X-ray crystallography. Several of these follow-up hits have affinities increased by more than one order of magnitude compared with the original fragment hit. The best case exhibited a 266-fold improvement in affinity. In conclusion, the presented approach can successfully identify commercially available follow-up candidates in one step, thereby circumventing laborious chemical synthesis in the early stage of fragment hit advancement.

## 2. Materials and methods   

### 2.1. Retrieval of commercially available fragment analogs   

Using the MolPort Chemical Search node (SIA MolPort, Latvia) within the Konstanz Information Miner (KNIME) version 3.4.0 (Berthold *et al.*, 2008 ▸), commercially available fragment analogs were retrieved. Three types of search were carried out: searching for analogs (i) that contain the initially discovered fragment as a substructure, (ii) that are a substructure of this fragment or (iii) that are reasonably similar to the corresponding fragment based on a MACCS fingerprint Tanimoto coefficient of ≥0.7 (Willett *et al.*, 1986 ▸). An increased upper limit of 10 000 retrievable structures per search type was granted by MolPort. Duplicate analogs were removed based on their MolPort IDs. Likewise, analogs containing atoms other than C, H, N, O, P, S, F, Cl, Br, I or Se were removed using the Chemistry Development Kit (CDK) Element Filter node (Beisken *et al.*, 2013 ▸). Very small molecules (molecular weight of <50 Da or containing less than four non-H atoms) were excluded from the similarity-search results based on calculations with the Standard Properties node of the LigandScout extensions for KNIME (Inte:Ligand GmbH). Also, a secondary similarity filter was applied requiring a Tanimoto coeffcient of ≥0.4 to the corresponding fragments using Indigo 2 structural fingerprints within KNIME (EPAM Systems Inc., Newtown, Pennsylvania, USA). Molecular formats were converted using the MolConverter node of ChemAxon LCC. The 3D conformers of the follow-up candidates were then generated by *OMEGA* (Hawkins *et al.*, 2010 ▸) version 2.5.1.4 from OpenEye Scientific Software.

### 2.2. Selection of EP–fragment complexes for optimization   

In order to test the intended optimization, five EP–fragment complexes (Table 1 ▸) were selected from the CFS campaign carried out by Radeva, Schiebel *et al.* (2016 ▸). The nomenclature of the starting fragments **F005**, **F041**, **F058**, **F066** and **F290** is defined as in Radeva, Schiebel *et al.* (2016 ▸). The follow-up compounds are named **FU_*x*_-*y***, where the subscript *x* denotes the respective starting fragment and *y* denotes the number of the follow-up compound of this series.

### 2.3. Preparation of receptors for docking of follow-up molecules   

Each fragment-bound EP structure was treated separately for docking and a separate list of analogs was retrieved. Here, only superstructures, *i.e.* structures containing the exact scaffold of the fragment as a substructure, of the used starting fragments were docked because at the time that the workflow was applied, to the best of our knowledge, no procedure was available to superimpose fragments with different scaffolds. Prior to the template-docking procedure developed in this work, the fragment-bound EP crystal structures were prepared manually with the *LeadIT* software (version 2.1.8), considering only amino-acid residues within 10 Å of the bound fragment and using default settings. Water molecules, fragments and other solutes were removed.

### 2.4. Scoring of docked poses   

For rescoring the binding poses after the customized *FlexX* template docking, DrugScoreX (DSX) was used (Neudert & Klebe, 2011*a* ▸). The program *DSX* can be downloaded freely from https://agklebe.pharmazie.uni-marburg.de/. DSX was chosen as it is somewhat tolerant of the close atomic contacts that may arise due to the geometric constraints of template docking to a rigid crystal structure. More specifically, the DrugScore (Gohlke *et al.*, 2000 ▸) per-contact score (PCS) is used from the DrugScore^PDB^ scoring function implemented in DSX (Neudert & Klebe, 2011*a* ▸). This PCS is the genuine DrugScore score divided by the number of atom–atom interactions within 6 Å of the ligand that contribute to the overall score. Thus, the PCS is a measure of interaction efficiency and sorting poses by PCS aims to enrich small but efficiently binding analogs that largely retain or improve the ligand efficiency (LE) of the corresponding fragment hit. In FBLD, a high LE is an indicator of well anchored fragments that bind efficiently with respect to their size and thus are good starting points for further optimization.

### 2.5. Protein purification and crystallization   

EP was isolated from Suparen (kindly provided by DSM Food Specialties, Heerlen, the Netherlands) in 0.1 *M* sodium acetate buffer pH 4.6 as described previously (Köster *et al.*, 2011 ▸). The sample was then subjected to size-exclusion chromatography using a Superdex S200 26/60 column (GE) and the same batch of buffer as for isolation. Protein-containing fractions were pooled, concentrated and flash-cooled in liquid nitrogen. The protein was then crystallized in a vapor-diffusion experiment in 48-well format using 250 µl reservoir solution consisting of 0.1 *M* sodium acetate pH 4.6, 0.1 *M* ammonium acetate pH 7.0, 24–33%(*w*/*v*) PEG 4000. 1.5 µl protein solution at a concentration of 5 mg ml^−1^ was mixed with an equal amount of reservoir solution. Trays were incubated at 20°C. Crystals appeared after 5–6 days and were then crushed using a seed-bead kit (Douglas Instruments) to prepare crystal seeds, which were then used in a second crystallization experiment, here using 27%(*w*/*v*) PEG 4000 in the reservoir and adding 0.1 µl of seed dilutions of 1:15–1:45 (seed stock:reservoir) to the freshly mixed drop of protein and reservoir. The seeded crystals appeared after three days.

### 2.6. Compound-soaking experiments   

The follow-up compounds (a full list, including providers and purities, if known, is given in Supplementary Table S1) were directly dissolved in a soaking solution consisting of 68.2 m*M* sodium acetate pH 4.6, 68.2 m*M* ammonium acetate pH 7.0, 16.9%(*w*/*v*) PEG 4000, 19.3%(*v*/*v*) glycerol, 9.09%(*v*/*v*) DMSO to a concentration of 100 m*M*. For poorly soluble follow-up compounds, crystals were soaked in the supernatant of the solution. After incubation for 16–22 h the crystals were flash-cooled in liquid nitrogen and stored for diffraction data collection.

### 2.7. Diffraction data collection and processing   

All data collections were carried out on beamlines BL14.1 and BL14.2 of the BESSY II electron-storage ring operated by the Helmholtz-Zentrum Berlin (HZB; Mueller *et al.*, 2015 ▸). Typically, 360° of data were collected in 0.1° increments using an exposure time of 0.1 s. Data were automatically processed using *XDSAPP* (Sparta *et al.*, 2016 ▸). All relevant data-collection and processing statistics are listed in Table 2 ▸.

### 2.8. Structure refinement and hit identification   

All structures were refined using the automated script *fspipeline*, which is based on Schiebel, Krimmer *et al.* (2016 ▸). The starting model was based on PDB entry 4y5l (Schiebel, Krimmer *et al.*, 2016 ▸), from which all ligands and water molecules were removed. Electron-density maps and coordinate files obtained from the automated refinement were inspected manually for each experiment to judge the presence or absence of the expected ligand in the difference electron density. Subsequently, the identified hits were subjected to several rounds of alternating model building in *Coot* (Emsley *et al.*, 2010 ▸) and refinement in *Phenix* (Liebschner *et al.*, 2019 ▸) before and after ligand placement. For all follow-up ligands, occupancy refinement was carried out. Refined models and the corresponding electron-density maps were submitted to the PDB under group deposition ID G_1002201. PDB codes for the single entries and all relevant structure-refinement and validation parameters are shown in Table 3 ▸.

### 2.9. Isothermal titration calorimetry (ITC)   

ITC experiments were conducted similarly to the procedure desribed by Schiebel, Radeva *et al.* (2016 ▸) on a MicroCal ITC200 (Malvern) instrument. All buffer solutions for ITC were prepared with the same batch of buffer as used to isolate the batch of EP. Details of the ITC experiments, including the protein and ligand concentrations for each experiment, are listed in Supplementary Table S2. In brief, the affinities of the weakly binding follow-up ligands were determined by displacement ITC titrations using the strongly enthalpic EP inhibitor SAP114 (Kuhnert *et al.*, 2015 ▸) as the displacement ligand. For this, 500 µ*M* SAP114 in a buffer solution consisting of 0.1 *M* sodium acetate pH 4.6, 3%(*v*/*v*) DMSO was titrated into the same buffer additionally containing 50 µ*M* EP and 2.0 m*M* of the respective follow-up ligand to a final stoichiometry of *N* = 2 (SAP114:EP). As a reference for calculating the affinities of the weakly binding follow-up ligands (Rühmann *et al.*, 2015 ▸), 500 µ*M* SAP114 was titrated into the buffer solution without follow-up ligand using the same protocol. All displace­ment titrations were conducted as single measurements, except for that of **FU_5_-2** (*n* = 3). The affinities of the stronger binding follow-up ligands were determined by direct ITC titrations. For this, 1 m*M* compound in a buffer solution consisting of 0.1 *M* sodium acetate pH 4.6, 3%(*v*/*v*) DMSO was titrated into the same buffer additionally containing 50 µ*M* EP to a final stoichiometry of *N* = 4. Due to the poor solubility of **FU_5_-1**, its affinity was determined in triplicate using the same protocol but titrating 500 µ*M* **FU_5_-1** against 25 µ*M* EP in the presence of 0.1%(*v*/*v*) Tween 20 in all buffers to a final stoichiometry of *N* = 4 (**FU_5_-1**:EP). For **FU_5_-1**, the available amount (1 mg) was used up by soaking experiments, so we resynthesized **FU_5_-1**·HCl (98.5% purity) for use in ITC experiments. Further details of the synthesis of **FU_5_-1**·HCl, including experimental data and NMR spectra, can be found in the supporting information (Supplementary Figs. S1–S3).

The obtained thermogram peaks of all titrations (Supplementary Fig. S4) were integrated with *Nitpic* 1.1.8 (Keller *et al.*, 2012 ▸). Subsequently, fitting of a single-site binding-model isotherm was performed using *SEDPHAT* 10.58d (Houtman *et al.*, 2007 ▸). For the errors of the fit, see Supplementary Table S3. For **FU_5_-1**·HCl, we used the *AFFINImeter* suite (version 2.1710; Muñoz & Piñeiro, 2018 ▸) to perform a global fit over three independent measurements to derive common values for *K*_a_ and Δ*H* (fits are also depicted in Supplementary Fig. S4). During the fit, Δ*H* was corrected for the heat of dilution, which was individually fitted for each experiment. The stoichiometry was arbitrarily fixed at the anticipated stoichiometry of *N* = 1 as appropriate for the present low *c*-value titrations (Rühmann *et al.*, 2015 ▸). The obtained goodness of fit was consistent for all three experiments (77.2%, 72.6% and 74.2%) and with the global goodness of fit (74.7%). Furthermore, the local minima table showed that the obtained fit was independent of the initial seed value of the algorithm in 20 independent rounds of fitting. Results from the global fit were comparable to those from individually fitting each experiment, yet were more robust in terms of numerical stability when using different seed values.

### 2.10. Restrospective and unbiased docking of crystallographically determined follow-up poses   

*SeeSAR* (version 11.0.0; BioSolveIT; license required) was used to prepare the receptors, perform the docking and score the resulting poses. For receptor preparation, the automatic pocket identification of *SeeSAR* was used on the complexes of **F005**, **F058**, **F066** and **F290** with EP (PDB codes are given in Table 1 ▸). The *FlexX* (Rarey *et al.*, 1996 ▸) functionality of *SeeSAR* was used for docking and a maximum number of poses of 500 was chosen. The docked poses were scored using the implemented HYDE scoring function (Reulecke *et al.*, 2008 ▸; Schneider *et al.*, 2013 ▸). The structural models of the follow-up compounds were aligned with the respective receptor structure. R.m.s.d.s of the scored poses versus the crystal structure pose of each follow-up were determined using *fconv* (Neudert & Klebe, 2011*b* ▸), which can be downloaded freely from https://agklebe.pharmazie.uni-marburg.de/.

## 3. Results   

### 3.1. Workflow for fragment growth using template docking   

**Figure 1:**
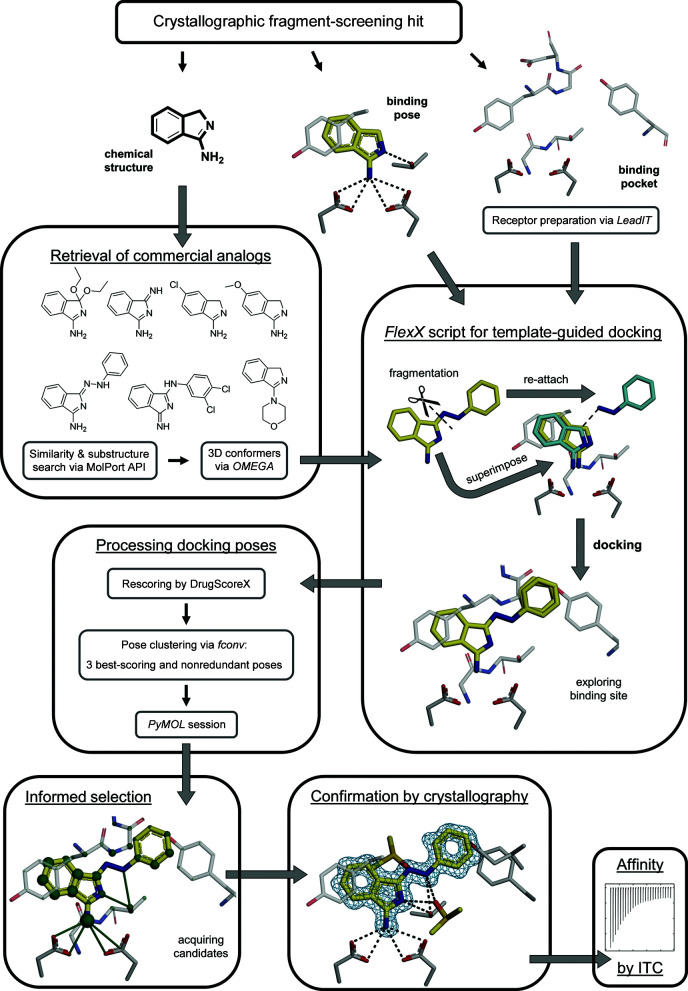
Frag4Lead workflow for fragment growing. The starting point of the workflow is a crystallographically detected fragment hit. It provides two types of information. The first is the identity, *i.e.* the chemical structure, of the fragment hit, based on which potential follow-up candidates are retrieved from the commercial catalog of MolPort and 3D conformers generated by *OMEGA*. The second is the 3D information of the binding pose of the fragment hit inside the binding pocket. The binding pocket is then prepared as a docking receptor via the *LeadIT* software (see Section 2.3 for details). Template-guided docking is then employed via a customized script using *FlexX* (Rarey *et al.*, 1996 ▸) using the crystallographic binding pose of the fragment as a starting point. Specifically, the *FlexX* algorithm cleaves each analog into internally rigid fragments (referred to as ‘FlexX fragments’). The FlexX fragment most similar to the starting fragment is then superimposed on the latter. Finally, each analog is incrementally reassembled by flexibly attaching its constituent FlexX fragments to the superimposed base fragment and the binding site is explored by *FlexX* docking. A maximum of 100 docking poses for each analog are generated and only the 1000 highest-scoring analogs are considered. In rare cases this process needs manual intervention, for example pruning of the docking template. The next vital step in the Frag4Lead workflow is the processing of the docking results. The docking poses generated by *FlexX* are rescored by the DrugScoreX per-contact score (see Section 2.4 for details). Next, redundant docking poses that are very similar to a better scored retained pose and would otherwise complicate the assessment of relevant poses are removed. To this end, the following procedure is applied to the docking poses of each analog. Firstly, all poses are clustered by hierarchical complete-linkage clustering with an r.m.s.d. threshold of 2.0 Å as implemented in *fconv* (Neudert & Klebe, 2011*b* ▸). Only the three best-scoring, nonredundant and internally sorted poses are kept. This efficiently eliminates redundant poses and allows the direct comparison of unique poses of each analog. In order to present the ranked hit list for interactive evaluation in a way that is also amenable to non-expert users, a *PyMOL* session is created that highlights the interactions and per-atom contributions (green spheres) to the overall DrugScoreX score of the pose. Unfavorable interactions and contributions are likewise highlighted. This enables a convenient and informed selection of follow-up compounds to be acquired based on the following criteria: (i) the ability of an analog to bind in the corresponding fragment pose, (ii) the location of most of its structure in a favorable environment, indicated by high but evenly distributed per-atom contributions to the overall DrugScoreX score, (iii) the formation of additional or alternative interactions compared with the starting fragment and (iv) the adoption of a realistic conformation. The binding of acquired compounds is then investigated by X-ray crystallography. The blue mesh shows the 2*mF*_o_ − *DF*_c_ electron-density map for the follow-up ligand contoured at σ = 1.0. Observed binders are then further evaluated by ITC to assess their binding affinity.

Elaborating fragment hits into more potent binders using commercially available compounds by exploiting the 3D structural information of the binding pose of a fragment is a very promising and at the same time a very cost-effective strategy in FBLD. Despite several advances and example campaigns, to the best knowledge of the authors this approach is not readily available as a routine or a (semi)-automated procedure. In order to fill this gap, such an optimization workflow was designed, developed and evaluated here. The different steps of the entire workflow, which is termed Frag4Lead, are presented graphically in Fig. 1 ▸. Based on a crystal structure of a fragment hit, structurally homologous compounds are retrieved from the catalog of commercially available compounds, in this case MolPort. For this, a con­venient search function either via a web interface or an application programming interface (for example the MolPort KNIME node) is employed. The next step and central part of the workflow utilizes the *FlexX* docking algorithm (Rarey *et al.*, 1996 ▸) to cleave analogs into ‘FlexX fragments’, which are then superimposed onto the crystallographically bound fragment. The FlexX fragment that best matches the template fragment structurally is then used as the ‘base fragment’ to reattach the remaining FlexX fragments in the environment of the binding pocket. In doing so, flexibly attaching moieties to the base fragment generates up to 100 docking poses so that thorough exploration of the binding pocket is ensured. Further on, the workflow includes a specific way to process and filter the docking results. For this, the *FlexX* docking poses were rescored by the DrugScoreX (DSX) per-contact score (Neudert & Klebe, 2011*a* ▸). Only high-scoring unique poses identified by r.m.s.d. clustering were retained and ranked. An informed selection of follow-up candidates was then performed in a *PyMOL* session (*PyMOL* version 2.0; Schrödinger), highlighting favorable contact distances, per-atom contributions to the overall DSX score and molecular properties that are relevant for FBLD. Selected follow-up candidates are then purchased and validated by soaking and crystallographic structure determination. Endothiapepsin crystals usually diffract to high resolution, which is certainly beneficial for identifying the exact binding pose of the compounds. The affinities of successfully confirmed follow-up compounds are then measured via ITC. In this way, one can complete an entire round of optimization without applying any chemistry or ordering customized synthesis.

### 3.2. Starting fragments and follow-up compounds   

**Figure 2:**
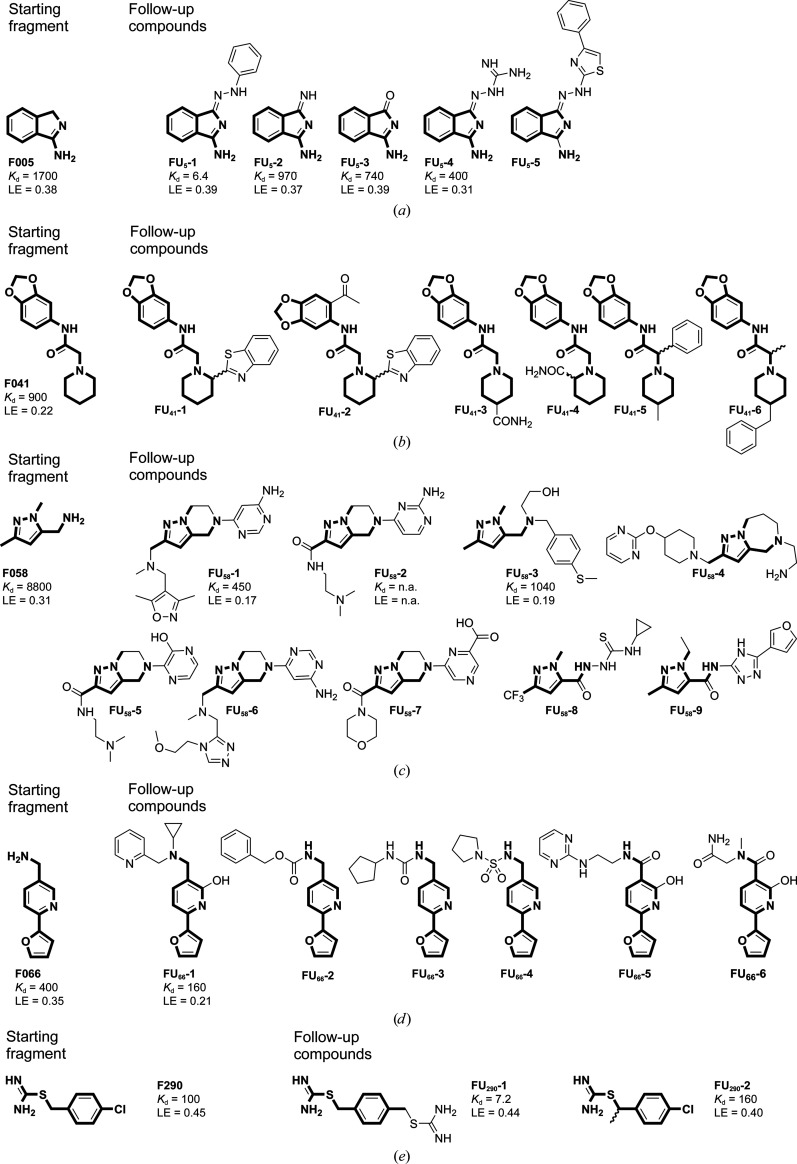
Starting fragments and follow-up compounds. The 2D chemical formulae of the five starting fragments of this work and the acquired follow-up candidates are given in (*a*)–(*e*). *K*_d_ is the dissociation constant of the compound from EP in µ*M* and LE is the respective ligand efficiency in kcal mol^−1^ per atom. All crystallographic binders were evaluated by ITC, except for **FU_58_-2**, where sufficient material for this purpose was not available. The *K*_d_ and LE values for the starting fragments were obtained in previous work (Schiebel, Radeva *et al.*, 2016 ▸).

In order to evaluate the power and success rate of Frag4Lead, five fragment hits that were previously discovered for EP (Radeva, Schiebel *et al.*, 2016 ▸) were used as starting points. Particular attention was paid to emulate a real-case scenario with only a few and potentially non-optimal fragment hits available and by testing only a limited number of commercially available follow-up candidates. Only about 25–30 follow-up compounds were aimed to be acquired in order to mimic an economically realistic scenario in a typical academic setting. Table 1 ▸ depicts the selected five starting fragments and the number of potential follow-up compounds retrieved from the catalog searches. Typically, such a search reveals several hundred potential follow-up compounds, and in this campaign between 267 and 10 022 compounds were obtained. These were then narrowed down to 28 compounds highly ranked by the docking, filtering and visual inspection in the Frag4Lead workflow. Fig. 2 ▸ lists all of the selected follow-up compounds of the five starting fragments.

### 3.3. Validating the fragment pose   

**Figure 3:**
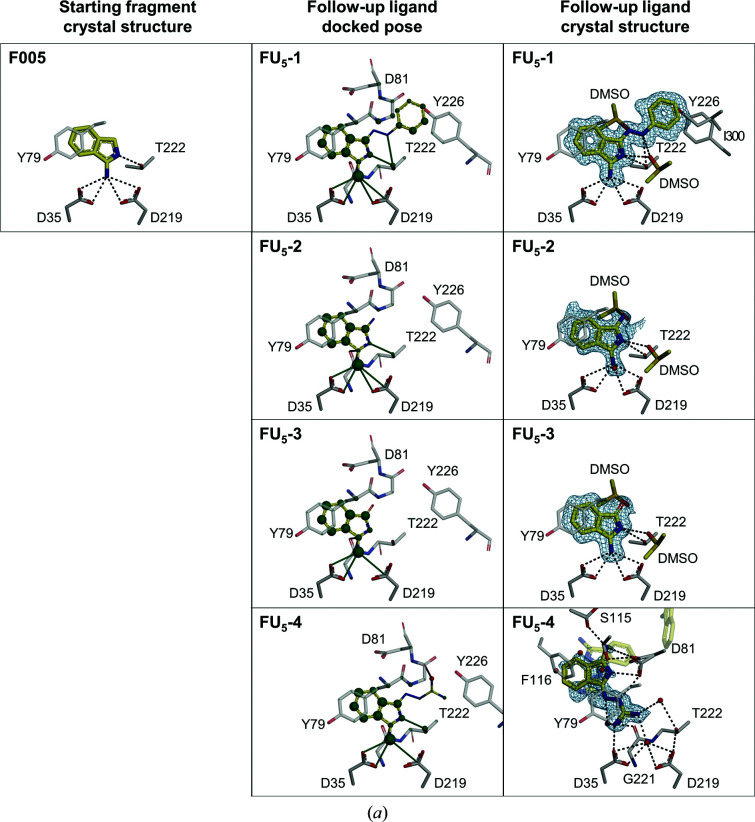

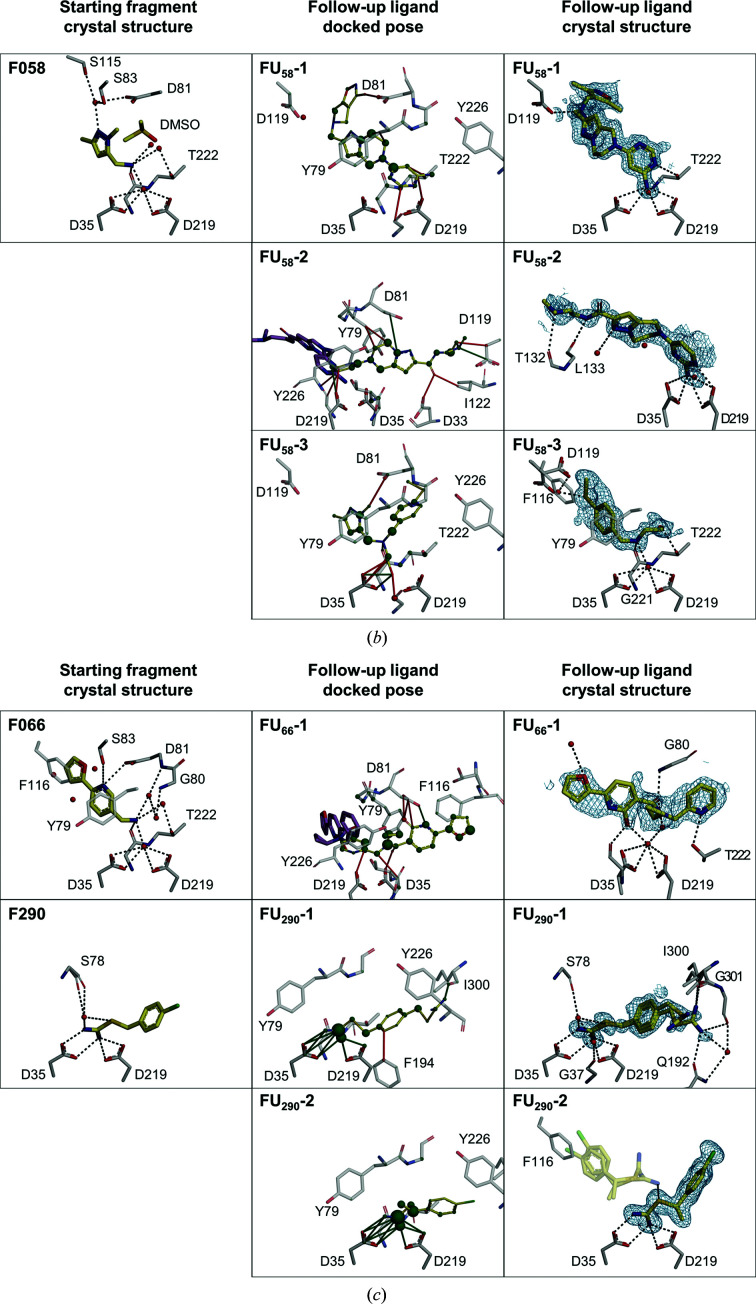
Side-by-side view of experimental and predicted binding poses. Shown are the binding poses of the starting fragments (left column), the docked poses of the follow-up ligands (middle column) and the binding poses of the follow-up ligands superimposed on polder OMIT *mF*_o_ − *DF*_c_ electron-density maps (Liebschner *et al.*, 2017 ▸) contoured at σ = 3.0 (right column) as observed in the crystal structures for all ten follow-up ligand structures. (*a*) Fragment **F005** and follow-up ligands. (*b*) Fragment **F058** and follow-up ligands. (*c*) Fragments **F066** and **F290** and the respective follow-up ligands. (*a*, *b*, *c*) For comparison of the docking poses to the original crystallographic fragment pose, all views are identical, except for **FU_58_-2** and **FU_66_-1**. For the latter, the crystallographic binding poses are also shown (purple sticks) to allow a comparison of the deviating binding poses. For the docking poses, favorable and unfavorable contact distances (green and red lines) and per-atom contributions to the overall DrugScoreX score (green and red spheres, with a radius approximating the score contribution), as predicted by *DSX* (Neudert & Klebe, 2011*a* ▸), are highlighted. For the crystal structures, polar interactions are shown as dashed lines. Ligands (yellow) and interacting residues (gray) are depicted as sticks with standard color-coding for heteroatoms and are labeled in single-letter code. Only primary binding poses near the catalytic dyad are depicted.

As a very first step in the optimization process, ideally even before employing the described workflow, it is important that the initial fragment-binding pose is thoroughly validated. This means that it needs to be assessed whether the binding pose observed by X-ray crystallography is retained for other highly similar analogs embedding the parent scaffold of the initial fragment hit, in order to minimize the risk of unexpected binding-mode changes during compound development. This is exemplified for starting fragment **F005** (Radeva, Schiebel *et al.*, 2016 ▸) and the follow-up compounds **FU_5_-2** and **FU_5_-3** (Fig. 3 ▸ *a*). **F005** binds to the catalytic center of EP and establishes charge-assisted hydrogen bonds to the two catalytic aspartate residues (Fig. 3 ▸ *a*). The two closely related analog fragments **FU_5_-2** and **FU_5_-3** differ from **F005** only by one additional atom at the 3 position. Crystal structure determination confirmed that these two follow-up fragments indeed retained the binding pose of **F005**. There are no additional directional interactions with the protein. However, in both structures an additional interaction with a DMSO molecule is observed which is not present in the original fragment **F005** structure. Strictly speaking, with such similar compounds a template-based docking approach is not needed. However, the docking was applied to all follow-up compounds irrespective of similarity and size. In this way, candidates are eliminated by the automated workflow if they contain minimal modifications of fragments that are incompatible with the binding mode, either sterically or due to mismatched interactions. This allows the identification of close analogs that are suitable for pose validation. However, in the subsequent rapid fragment growing performed in this work, the other four starting fragments were not as stringently subjected to an experimental validation step as **F005** and were more directly used for elaboration with the objective of fast affinity improvement (Table 1 ▸).

### 3.4. Applying the Frag4Lead workflow to EP   

Table 1 ▸ lists the potential follow-up compounds that could be found in the catalog for each of the five starting fragments and the number that are left after template-based docking has been applied as a filter. Typically, template-based docking reduces the number of candidate follow-up molecules by roughly one order of magnitude from several hundred to several dozen candidates. These were inspected visually in *PyMOL*. Based on this, 28 follow-up candidate molecules were selected and acquired for further testing by X-ray crystallography. Successful binders were subjected to ITC in order to retrieve information about the improvement in affinity compared with the starting fragment (Fig. 2 ▸). In the next paragraphs the crystallographic results will be described in detail and in the context of the obtained affinity measurements, grouped by the respective starting fragments.

### 3.5. Follow-up compounds for starting fragment **F005**   

**Figure 4:**
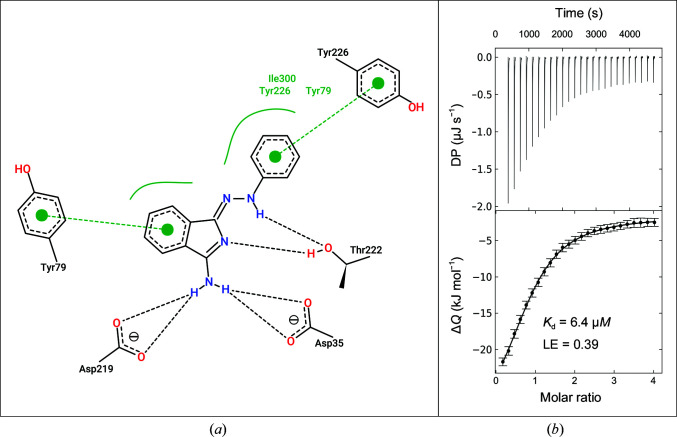
Details of the interaction of **FU_5_-1** with EP and the corresponding ITC results. (*a*) For the highest affinity binder identified with the applied workflow, **FU_5_-1**, the atomic interaction network is shown. The picture was generated with *PoseView* (Stierand & Rarey, 2010 ▸). (*b*) Representative ITC thermogram of the direct titration of **FU_5_-1** against EP.

For **F005** five follow-up candidates were selected (Fig. 2 ▸ *a*), four of which were observed in crystal structures (Fig. 3 ▸ *a*). The strongest affinity improvement was obtained with **FU_5_-1**. In this case, the pose of the starting fragment is retained (r.m.s.d. = 0.41 Å) and the additional phenylhydrazone group led to a 266-fold affinity increase from 1.7 m*M* to 6.4 µ*M*, while maintaining the ligand efficiency (LE). **FU_5_-1** is a rigid molecule that does not interfere with the geometry of the binding pocket. Consequently, **FU_5_-1** binds while maintaining its minimal energy conformation in the protein environment. The affinity increase of **FU_5_-1** compared with **F005** is accompanied by the following additional interactions. **FU_5_-1** forms a hydrogen bond between its hydrazone NH group and the hydroxyl O atom of Thr222 (*d*_N—H⋯O_ = 3.1 Å). Furthermore, the phenyl ring of **FU_5_-1** forms hydrophobic and π-stacking interactions with the side chain of Tyr226, the amide bonds of Gly80 and Asp81, and the side chain of Ile300 (Fig. 4 ▸ *a*). The latter undergoes an induced fit to contact **FU_5_-1**, concomitantly stabilizing the adjacent sequence segment (Ala298–Ile302). Compared with the **F005** complex, **FU_5_-1** displaces no additional structural water molecules, yet it is in close contact with two DMSO molecules recruited to the binding site. Each of these DMSO molecules displaces a structural water molecule present in either the **F005** complex or the apo structure. Apparently, there is no well formed hydrogen bond between **FU_5_-1** and the DMSO molecules. Even though the O atom of a DMSO molecule is close to the hydrazone NH group (*d*_N—H⋯O_ = 3.1 Å), both form a non-ideal angle of β(N—H⋯O) = 120° and the NH group of **FU_5_-1** already forms a hydrogen bond to Thr222. Thus, as expected, soaking in the absence of DMSO did not alter the pose of **FU_5_-1**; the originally present DMSO binding site turns out to be occupied by an acetate ion from the buffer instead (data not shown). This suggests that DMSO, which had to be included in the ITC experiments for all ligands for sufficient solubilization, does not alter the apparent affinity of **FU_5_-1**. The follow-up compounds **FU_5_-2** and **FU_5_-3** have already been described above. They each differ from **F005** merely by one atom, which does not engage in any new hydrogen bonds. Also, **FU_5_-2** and **FU_5_-3** exhibit nearly the same dissociation constant and ligand efficiency values as **F005**. Hence, the additional atom also does not seem to influence the strength of the hydrogen bonds compared with the **F005**–EP complex. **FU_5_-4**, however, is surprisingly bound in a reversed orientation, forming a salt bridge to Asp81 via its isoindole N atom while its aminoguanidine moiety forms a salt bridge to the catalytic dyad that is partially mediated by the catalytic water. A similar binding mode to the catalytic dyad was found in an earlier screening for fragments bound via their guanidine and amidine groups, but none of them utilized the catalytic water (Radeva, Schiebel *et al.*, 2016 ▸). It may be speculated that the strong interaction between the additional guanidinium group and the catalytic dyad led to the reversal of the orientation of **FU_5_-4**. This allegedly non-optimal pose of **FU_5_-4** is accompanied by only a slight increase in affinity (*K*_d_ = 400 µ*M*) and by a significant decrease in LE (LE = 0.31 kcal mol^−1^ per atom). A possible explanation for the minor affinity enhancement could be a presumably strong increase in the desolvation costs of this more polar fragment upon binding. However, conclusive reasoning in the case of such large changes of binding mode is difficult in general.

### 3.6. Follow-up compounds for starting fragment **F041**   

For **F041** six follow-up candidates were selected (Fig. 2 ▸ *b*), none of which was observed in a crystal structure. It may be the case that the low ligand efficiency of **F041** (LE = 0.22 kcal mol^−1^ per atom) already indicated weak binding, and a preceding binding-pose validation using closer analogs would have been highly advisable.

### 3.7. Follow-up compounds for starting fragment **F058**   

For **F058** nine follow-up candidates were selected (Fig. 2 ▸ *c*), which is the largest number for all five starting fragments in this work. Three of them (**FU_58_-1**, **FU_58_-2** and **FU_58_-3**) were observed in crystal structures (Fig. 3 ▸ *b*), but none of them maintained the binding pose of the original fragment. However, **FU_58_-1** bound with the corresponding portion still in the proximity of the original position of **F058** in the S1 pocket. The diazole ring is flipped and located roughly two bond lengths further away from the catalytic dyad. Notably, this shift enables the formation of a salt bridge between the 4-aminopyrimidine moiety of **FU_58_-1** and the catalytic dyad, with the amino N atom displacing the catalytic water. Additionally, a salt bridge is formed from the tertiary amine of **FU_58_-1** to the carboxylate O atom of Asp119 (*d*_N—H⋯O_ = 2.9 Å). However, the intricate network of water-mediated interactions between **F058** and Asp81, Ser83, Ser115 and Thr222, as well as the catalytic dyad, was largely not formed in the **FU_58_-1**–EP complex, most likely due to the missing primary amine of **F058**, which in **FU_58_-1** was replaced by a tertiary amine connecting to the 4-aminopyrimidine moiety. **FU_58_-1** exhibits an about 20-fold higher affinity than the weakly bound **F058** in an ITC experiment (*K*_d_ = 450 µ*M* *versus* 8.8 m*M*), but it also contains 17 more non-H atoms than the starting fragment. Consequently, the ligand efficiency is decreased drastically compared with **F058** (LE = 0.17 *versus * 0.31 kcal mol^−1^ per atom). Similarly, **FU_58_-2** binds displacing the catalytic water with its 4-aminopyrimidine moiety, yet mirrored at the plane spanned by the carboxylate groups of the catalytic dyad. Thus, the remainder of **FU_58_-2** is oriented in the S1′ direction, also occupying the S2′ pocket of the substrate-binding cleft. Here, **FU_58_-2** forms two direct and one water-mediated hydrogen bonds in addition to the salt bridge with the catalytic dyad, yet it does not form a salt bridge via its terminal tertiary amine. Unfortunately, **FU_58_-2** could not be characterized by ITC because sufficient material was not available. In the structure obtained by soaking **FU_58_-3**, only its substructure analog **FU_58_-3b** (2-{[4-(methylthio)benzyl]­amino}ethan-1-ol) could unambiguously be identified in the electron density and built into the crystal structure after verifying its presence as an impurity in the obtained sample of **FU_58_-3** (purity of >90% according to the provider) by mass spectrometry (see supporting information, including Supplementary Fig. S5). Given the lack of electron density for **FU_58_-3** in our crystallographic experiment, it may be speculated that either **FU_58_-3** does not bind in solution as well or that **FU_58_-3b** efficiently competes with **FU_58_-3** in the crystal structure. One might also speculate that the true concentration of the active species in the displacement ITC experiment is underestimated, so that the apparent *K*_d_ of 1040 µ*M* must be considered an upper limit. However, despite the identification of **FU_58_-3b** by mass spectrometry, the presence of other, potential nonspecific species in the impure sample cannot be excluded, so that attributing the apparent *K*_d_ to any specific compound is highly unreliable.

### 3.8. Follow-up compounds for starting fragment **F066**   

For **F066** six follow-up candidates were selected (Fig. 2 ▸ *d*), of which one was observed in the crystal structure (Fig. 3 ▸ *c*). **FU_66_-1** did not maintain the original binding pose observed for **F066**. Instead, it bridges the catalytic dyad, thereby accessing both directions of the peptide-binding cleft. This new pose is facilitated by the additional hydroxyl group of **FU_66_-1**, which is located vicinal to the pyridine N atom of **F066**. Although this additional hydroxyl group was predicted to be compatible with the fragment pose, it unexpectedly forms new hydrogen bonds to the catalytic water as well as the carbonyl O atom of Gly80 in the pose of **FU_66_-1**.

### 3.9. Follow-up compounds for starting fragment **F290**   

**F290** is a special case for follow-up candidate selection. Compounds that contain an isothiourea moiety as part of a ring were not properly matched to the starting fragment. This problem was solved by pruning **F290** down to its isothiourea moiety for follow-up compound identification. In this way, two follow-up candidates were selected (Fig. 2 ▸ *e*), both of which were observed in crystal structures and maintained the original binding pose (Fig. 3 ▸ *c*). In addition, for both a second alternative binding pose was observed. The affinity of **FU_290_-1** is increased 14-fold (*K*_d_ = 7.2 µ*M*) compared with **F290** (*K*_d_ = 100 µ*M*). At the same time, the LE was left essentially unchanged (0.44 and 0.45 kcal mol^−1^ per atom, respectively). This means that the affinity of **FU_290_-1** increased proportional to its size. Thus, **FU_290_-1** may be another good starting point for further optimization, although the affinity determination was hampered by a noisy baseline in ITC experiments (Supplementary Fig. S4), allegedly due to its low purity (>90% according to the provider). The primary binding site of **FU_290_-1** is occupied by two conformers, which bind very similarly to **F290**. While conformer *A* [r.m.s.d. of the maximum common substructure (r.m.s.d._MCS_) = 0.20 Å, 42% occupancy] forms no additional direct polar interactions, conformer *B* (r.m.s.d._MCS_ = 0.29 Å, 53% occupancy) donates two additional hydrogen bonds from its guanidine NH group to the side-chain amide O atom of Gln192 (*d*_N—H⋯O_ = 3.3 Å) and to the equidistant backbone carbonyl O atom of Ile300 (*d*_N—H⋯O_ = 3.4 Å), which also adopts two alternative conformations. Soaking in racemic **FU_290_-2** resulted in (*R*)-**FU_290_-2** bound with the isothiourea moiety closely maintaining the pose in **F290**. However, the affinity was unchanged (*K*_d_ = 160 µ*M* for the racemic mixture) and the additional methyl group at the stereocenter coincides with a shift of the *p*-chlorobenzyl moiety away from its original position (r.m.s.d._MCS_ = 2.9 Å) towards the flap loop. This could be due to a steric clash or alteration of the torsional preference within **FU_290_-2**. The flap loop itself is displaced as well, and presumably this is the reason why docking did not produce the correct pose even before filtering. In addition, a nearby secondary site is weakly occupied by overlapping poses of (*R*)- and (*S*)-**FU_290_-2**, both of which form a π-stacking interaction with Phe116, while one donates a weak hydrogen-bond to the isothiourea S atom of the primary fragment.

All in all, the workflow assembled and tested here for filtering commercially available analogs of fragment hits via template-based docking proved to be successful in the EP campaign. From only five starting fragments and a limited number of 28 follow-up compounds acquired, ten binders were identified by crystallography. Five of the follow-up binders bound in the pose of the original fragment and four of them exhibited a significantly increased affinity. Two of them, **FU_5_-1** and **FU_290_-1**, even reached single-digit micromolar affinity and **FU_5_-1** showed a remarkable 266-fold improvement in affinity.

## 4. Discussion   

Fragment screening by crystallography typically provides multiple fragment hits as potential starting points for FBLD. For each promising hit, it is advisable to first test close analogs in order to validate the binding pose of a given fragment hit. In a next step, the fragment needs to be grown into a larger molecule with substantial affinity improvement. This is still the most challenging step in FBLD (for a review of such methods, see de Souza Neto *et al.*, 2020 ▸). One approach that ensures rapid progress of the project, especially in a typical academic setting with limited financial resources, is to exploit follow-up candidates that are readily available via vendor catalog databases. This limits the number of molecules compared with exploring large virtual chemical spaces, but still returns too many for the manual selection of promising compounds. Completely unbiased docking may help in an automated fashion, although this often generates binding poses that deviate from the original fragment, thus contradicting the idea of rational fragment-based design and complicating the comparison of the suggested poses.

Here, we demonstrate our Frag4Lead workflow, which is based on template-based virtual screening of commercially available follow-up compounds. It utilizes the fragment pose found in a crystal structure, for example from a crystallo­graphic fragment-screening campaign, as additional information. Frag4Lead was validated on the model system EP using a limited number of both starting fragments and compounds to be acquired. Of the more than 70 fragment hits identified by CFS against EP (41 addressing the catalytic dyad), five were selected for this study (Köster *et al.*, 2011 ▸; Radeva, Schiebel *et al.*, 2016 ▸; Radeva, Krimmer *et al.*, 2016 ▸). These five starting fragments were selected to emulate a real-case scenario with only a few and potentially non-optimal fragment hits to follow up on. From the five starting fragments, 28 follow-up compounds were identified and purchased. Out of the 28 selected follow-up compounds, ten binders could be identified. Even though the original fragment pose was retained for only five of them, two follow-up compounds exhibited a very successful advancement to an affinity of less than 10 µ*M*. An earlier study of docking-supported fragment growth performed similarly (Marchand *et al.*, 2016 ▸). There, six out of 16 selected candidates were binders (*i.e.* a similar success rate). However, the best affinity of 279 µ*M* reached is two orders of magnitude lower than in the campaign presented here. Also, compared with alternative approaches for the rapid elaboration of fragment hits, for example by screening diverse fragment follow-up compounds in crude reaction mixtures from fast chemistry (Baker *et al.*, 2020 ▸; Bentley *et al.*, 2020 ▸), the presented example campaign via Frag4Lead ended up with a similar number of hits and better affinity improvement. It seems rather obvious that these approaches could complement each other. For instance, a relatively large virtual chemical space of close fragment analogs with suitable reaction handles combined with building blocks available in-house could be constructed and filtered by template docking to identify the most promising candidates and the building blocks required for their synthesis.

The follow-up compound with the highest affinity in the campaign presented here, **FU_5_-1**, seems to be suitable for further ligand development for two reasons. Firstly, it forms a tight cluster of interactions, with the starting fragment substructure acting as an anchor. In addition, this anchor has an excellent growth vector along which the phenylhydrazone moiety of **FU_5_-1** is oriented, forming additional hydrogen bonds and π-stacking interactions with residue Tyr226. Most importantly, however, the simple and fast synthesizability of **FU_5_-1** and derivatives thereof (see supporting information) enables efficient exploration of this growth vector, thus making a rapid elaboration of possible interactions and structure–activity relationships feasible. Yet, despite its low micromolar affinity and favorable interactions, it may seem questionable whether the hydrazone structure of **FU_5_-1** is suitable for drug development. Reportedly, hydrazones may form hydrazines and other reactive or toxic derivatives (Smith, 2011 ▸). Indeed, decomposition of the synthesized **FU_5_-1** was observed when the compound was exposed to air at room temperature over a longer period of time. Nonetheless, **FU_5_-1** unambiguously bound in the crystal structure after soaking for 24 h at 18°C under slightly acidic conditions, indicating the stability of its protonated form in solution. Moreover, the existence of bioactive hydrazones, some of which are approved drugs (Rollas & Küçükgüzel, 2007 ▸), and the potential for bioisosteric replacement of the hydrazone, for example by amides or ureas, demonstrates that **FU_5_-1** and its derivatives may well be reasonable starting points for the development of lead or tool compounds.

Other follow-up compounds in the presented EP campaign did not maintain the anticipated binding pose. In fact, a change of binding mode upon chemical variation is not uncommon, and adding substitutions that enable new but competing interactions is reportedly a major cause of this (Malhotra & Karanicolas, 2017 ▸; Oebbeke *et al.*, 2021 ▸). In the case of **FU_5_-4**, for example, the changed binding pose could supposedly have been anticipated or predicted, as interactions of the guanidine moiety with the catalytic dyad are very plausible. Also, other fragments with a guanidine moiety were found to bind to the catalytic dyad, for example PDB entry 4ycy (Radeva, Schiebel *et al.*, 2016 ▸). One may also test for such possibilities via the template-docking approach in order to assess whether follow-up candidates are also compatible with the poses of other known fragment hits. However, approaches to estimate the absolute and relative stability of binding poses are difficult and laborious, so that crystallographic verification is often easier and more straightforward in the presence of a suitable crystal system and soaking condition. Therefore, these findings strongly encourage the validation of binding poses of fragment hits from a primary crystallographic screening using close analogs prior to embarking on growth strategies such as Frag4Lead.

Another follow-up ligand that did not maintain the binding pose of the starting fragment is **FU_58_-1**. This may not be surprising because although **FU_58_-1** is a superstructure of **F058**, its primary amino group, which forms a direct hydrogen bond to the catalytic water, is replaced by a tertiary anilinic nitrogen, thus losing its hydrogen-bonding capacity. Instead, the additional 4-aminopyrimidine moiety of **FU_58_-1** was anticipated to replace the catalytic water and address the catalytic dyad directly (although not observed in the docking pose). Indeed, this interaction was observed in the crystal structure but required a flip of the central heterocycle as well as a slight shift away from the catalytic dyad. However, in the docked pose of **FU_58_-1** the amino group pointing away from the catalytic dyad could have been interpreted as an indicator of a suboptimal interaction geometry of the central part of the ligand. For this reason, future improvements of the Frag4Lead workflow should aim at identifying unstable predicted binding poses in order to focus on the most promising starting fragments and their respective follow-up candidates.

One approach for future improvements may come from a better assessment of observed and predicted interactions, for example via descriptors based on the statistical occurrence of protein–ligand contacts in the PDB (Tosstorff *et al.*, 2020 ▸). Another improvement would be to predict the unexpected or flipped poses that were observed in the crystallographic experiments of the chosen follow-ups (*i.e.* **FU_5_-4**, **FU_58_-1**,** FU_58_-2**, **FU_58_-3** and **FU_66_-1**) with high confidence in order to deprioritize those compounds in the selection process. However, in a retrospective, unbiased docking experiment of the successful crystallographic binders using the newest GUI version of *SeeSAR* (version 11.0.0), unbiased *FlexX* docking and HYDE scoring did not produce any pose within an r.m.s.d. of 2 Å of the experimentally observed unexpected poses. Additionally, for the follow-up compounds that retained the binding pose of the fragment, none of the predictions turned up within the three highest scored poses. Only the pose of **FU_290_-2** showed up in the ten highest scored poses (see Supplementary Table S4). This again underlines the advantage of the template-based docking employed in the Frag4Lead workflow, making use of the obtained structural information of the fragment hits to improve the follow-up compound selection.

In view of the large and constantly growing space of reliably synthesizable compounds (van Hilten *et al.*, 2019 ▸), the presented template-guided docking approach enables the rapid early discovery of improved ligands without custom synthesis requirements. In addition, the underlying docking functionality has recently been developed and implemented similarly in the *SeeSAR* software (BioSolveIT GmbH) with further improved substructure-matching algorithms that allow the guided docking of close non-substructure analogs. However, for large virtual chemical spaces with billions of compounds, the computational cost will increase. This might require more efficient prefiltering to remove sterically incompatible follow-up candidates prior to docking, for example by employing the recently described shape-based descriptors (Penner *et al.*, 2020 ▸).

The presented generic strategy is able to identify suitable follow-up candidates from any source of analogs to exploit fragment-bound structures. Supposedly, it will be more efficient in combination with fragment libraries that are designed to comprise starting points for the easy exploration of large chemical spaces (Cox *et al.*, 2016 ▸). However, the presented concept also harmonizes with our newly introduced, structurally diverse F2X-Universal Library, which is based on 3D shape and pharmacophore clustering of a large, readily available fragment space and achieves high hit rates (Wollenhaupt *et al.*, 2020 ▸). For each member, *i.e.* cluster representative, of the F2X-Universal Library, there is a high likelihood that similar and readily purchasable compounds exist.

The Frag4Lead workflow evaluated here serves as a first attempt to automate initial fragment-hit expansion for non-expert users and projects with limited resources for laborious follow-up chemistry. This limitation is even more pronounced in academic settings and often provides the most critical bottleneck in academic compound development. A key reason for this is that funding for professional compound synthesis is much harder to acquire than for personnel and equipment. However, saving costs or ensuring faster progress through more efficient fragment expansion is also highly desirable in an industrial setting.

Clearly, the concepts employed in the Frag4Lead workflow need to be optimized further, in particular with respect to transferability to different sites. For now, the Frag4Lead workflow is available to all users of the HZB fragment screening facility. However, its successful application demonstrates its clear potential to contribute to more efficient structure-based ligand design, especially in academia, in the initial stage of drug development.

## 5. Related literature   

The following references are cited in the supporting information for this article: Biitseva *et al.* (2013 ▸), Krimmer & Klebe (2015 ▸) and Wolf & Vollmann (1956 ▸).

## Supplementary Material

PDB reference: endothiapepsin, 5sak

PDB reference: 5sal

PDB reference: 5sam

PDB reference: 5san

PDB reference: 5sao

PDB reference: 5sap

PDB reference: 5saq

PDB reference: 5sar

PDB reference: 5sas

PDB reference: 5sat

Supporting Information, Supplementary Tables and Supplementary Figures. DOI: 10.1107/S2059798321008196/ud5027sup1.pdf

## Figures and Tables

**Table 1:** EP–fragment complexes chosen for optimization The fragment nomenclature was adopted from Köster *et al.* (2011 ▸). *K*_d_ is the dissociation constant of the compound from EP and LE is the respective ligand efficiency, which is the binding energy per non-H atom. The *K*_d_ and LE values are taken from Schiebel, Radeva *et al.* (2016 ▸). The number of successfully docked analogs refers to docked analogs for which *FlexX* generated a meaningful pose.

Fragment	PDB code	Chemical structure	*K*_d_ (µ*M*)	LE (kcal mol^−1^ per atom)	No. of identified follow-up candidates	No. of successfully docked follow-up candidates
**F005**	4y3e	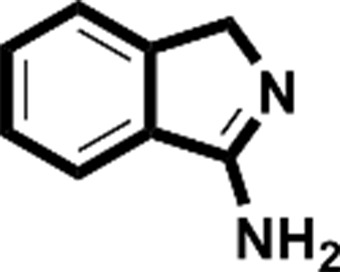	1700	0.38	556	67
**F041**	4y3z	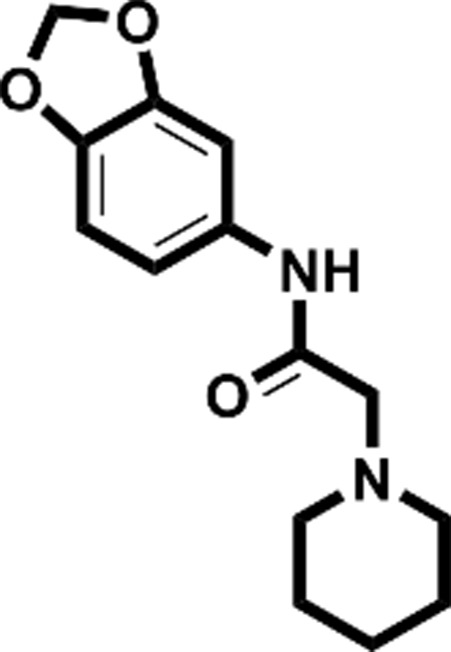	900	0.22	1013	88
**F058**	4y56	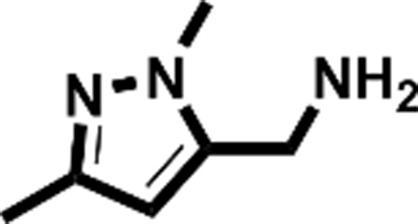	8800	0.31	10022	>1000†
**F066**	5dq4	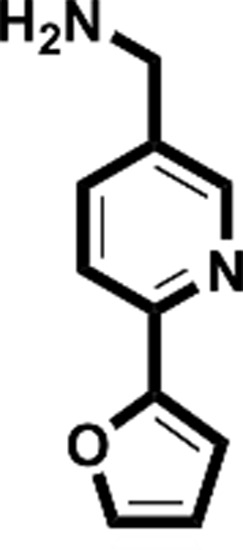	400	0.35	615	395
**F290**	4y35	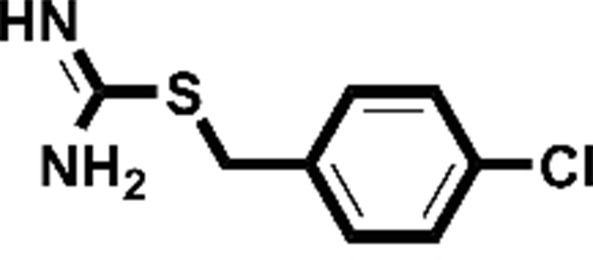	100	0.45	267	32

† Only the 1000 highest-ranking fragment analogs were considered.

**Table 2:** Data-collection and processing statistics Values in parentheses are for the outer shell.

Ligand ID	**FU_5_-1**	**FU_5_-2**	**FU_5_-3**	**FU_5_-4**	**FU_58_-1**	**FU_58_-2**	**FU_58_-3**	**FU_290_-1**	**FU_290_-2**	**FU_66_-1**
PDB code	5sak	5sal	5sam	5san	5sao	5sap	5saq	5sar	5sas	5sat
X-ray source	BESSY II	BESSY II	BESSY II	BESSY II	BESSY II	BESSY II	BESSY II	BESSY II	BESSY II	BESSY II
Beamline	BL14.1	BL14.1	BL14.1	BL14.1	BL14.1	BL14.1	BL14.1	BL14.1	BL14.1	BL14.1
Wavelength (Å)	0.9184	0.9184	0.9184	0.9184	0.9184	0.9184	0.9184	0.9184	0.9184	0.9184
Detector	PILATUS 6M	PILATUS 6M	PILATUS 6M	PILATUS 6M	PILATUS 6M	PILATUS 6M	PILATUS 6M	PILATUS 6M	PILATUS 6M	PILATUS 6M
No. of crystals	1	1	1	1	1	1	1	1	1	1
Temperature (K)	100	100	100	100	100	100	100	100	100	100
Detector distance (mm)	149.208	149.196	210.510	142.610	165.251	165.237	174.598	142.604	165.249	210.509
Rotation range per image (°)	0.1	0.1	0.1	0.1	0.1	0.1	0.1	0.1	0.1	0.1
Total rotation range (°)	360	360	360	360	360	360	360	360	360	360
Space group	*P*2_1_	*P*2_1_	*P*2_1_	*P*2_1_	*P*2_1_	*P*2_1_	*P*2_1_	*P*2_1_	*P*2_1_	*P*2_1_
Unit-cell parameters										
*a* (Å)	45.33	45.34	45.34	44.96	45.31	45.41	45.43	45.23	45.30	45.17
*b* (Å)	73.69	73.50	73.27	72.61	72.91	73.49	73.38	73.15	73.06	73.40
*c* (Å)	52.74	53.12	52.97	51.63	52.62	53.15	53.06	52.77	52.64	52.56
α (°)	90.0	90.0	90.0	90.0	90.0	90.0	90.0	90.0	90.0	90.0
β (°)	109.70	110.21	109.76	108.61	109.78	110.15	109.90	109.53	109.45	109.37
γ (°)	90.0	90.0	90.0	90.0	90.0	90.0	90.0	90.0	90.0	90.0
Resolution range (Å)	42.68–1.10 (1.17–1.10)	42.55–1.00 (1.06–1.00)	42.67–1.20 (1.27–1.20)	48.93–0.94 (1.00–0.94)	42.64–1.00 (1.06–1.00)	42.63–1.04 (1.10–1.04)	42.72–1.02 (1.08–1.02)	42.62–0.98 (1.04–0.98)	42.72–1.17 (1.24–1.17)	49.59–1.60 (1.70–1.60)
Total No. of reflections	468582	638330	338920	711675	611157	574223	586735	667615	401189	144529
Unique reflections	127768	173459	99863	198823	158879	152537	159315	175062	107431	42081
Multiplicity	3.67	3.68	3.39	3.58	3.85	3.76	3.68	3.81	3.73	3.43
Mean *I*/σ(*I*)	8.9 (0.7)	7.6 (0.8)	10.8 (0.9)	8.4 (0.7)	13.6 (1.1)	8.2 (0.9)	7.7 (0.8)	5.5 (0.8)	9.6 (0.8)	14.0 (1.9)
*R*_meas_ (%)	8.2 (172.0)	8.8 (143.3)	7.8 (136.8)	7.4 (157.7)	4.7 (117.9)	8.6 (137.3)	9.5 (130.3)	13.5 (121.7)	8.0 (164.5)	6.9 (73.3)
Completeness (%)	97.9 (93.2)	98.4 (97.8)	98.0 (97.0)	97.6 (91.8)	91.6 (78.0)	97.1 (95.0)	95.8 (85.7)	94.4 (90.9)	98.0 (95.1)	98.3 (98.1)
Wilson *B* factor (Å^2^)	16.19	13.16	17.16	12.03	13.87	13.60	12.73	12.12	17.56	24.79
Mosaicity (°)	0.137	0.055	0.157	0.072	0.083	0.093	0.062	0.053	0.135	0.341
CC_1/2_	99.9 (31.6)	99.7 (35.6)	99.9 (38.8)	99.7 (40.2)	99.9 (58.4)	99.8 (41.1)	99.7 (40.9)	98.7 (46.6)	99.9 (36.6)	99.9 (30.9)
ISa	21.6	14.6	39.2	16.6	30.3	17.8	15.8	7.8	23.1	37.7

**Table 3:** Structure-refinement and validation statistics Values in parentheses are for the outer shell. Refinement was performed with *phenix.refine* version 1.19.

Ligand ID	**FU_5_-1**	**FU_5_-2**	**FU_5_-3**	**FU_5_-4**	**FU_58_-1**	**FU_58_-2**	**FU_58_-3**	**FU_290_-1**	**FU_290_-2**	**FU_66_-1**
PDB code	5sak	5sal	5sam	5san	5sao	5sap	5saq	5sar	5sas	5sat
Resolution limits (Å)	42.68–1.10 (1.13–1.10)	42.55–1.00 (1.02–1.00)	42.67–1.20 (1.23–1.20)	48.93–0.94 (0.96–0.94)	42.64–1.00 (1.02–1.00)	42.63–1.04 (1.06–1.04)	41.26–1.02 (1.04–1.02)	41.13–0.98 (1.00–0.98)	42.72–1.17 (1.19–1.17)	49.59–1.40 (1.42–1.40)
Completeness (%)	98.0	98.5	98.1	97.6	91.6	97.2	95.9	94.4	98.1	98.1
Data cutoff	*F* > 1.33σ(*F*)	*F* > 1.33σ(*F*)	*F* > 1.35σ(*F*)	*F* > 1.35σ(*F*)	*F* > 1.36σ(*F*)	*F* > 1.35σ(*F*)	*F* > 1.35σ(*F*)	*F* > 1.36σ(*F*)	*F* > 1.35σ(*F*)	*F* > 1.35σ(*F*)
No. of reflections										
Working set	125640 (7260)	171349 (11272)	97759 (6355)	196698 (10913)	156756 (7204)	150436 (9664)	157202 (8192)	172802 (10946)	105317 (6461)	59494 (2686)
Test set	2101 (122)	2101 (138)	2101 (137)	2100 (116)	2101 (97)	2101 (135)	2100 (109)	2098 (133)	2100 (129)	3132 (141)
*R*_work_	0.143 (0.3678)	0.144 (0.3424)	0.138 (0.2883)	0.139 (0.4070)	0.131 (0.3680)	0.134 (0.3391)	0.139 (0.3717)	0.164 (0.3784)	0.140 (0.3328)	0.161 (0.3104)
*R*_free_	0.159 (0.3818)	0.160 (0.3579)	0.164 (0.2800)	0.147 (0.4078)	0.146 (0.3764)	0.149 (0.3445)	0.157 (0.3809)	0.175 (0.3676)	0.156 (0.3391)	0.205 (0.3315)
No. of non-H atoms										
Protein	2389	2389	2389	2389	2389	2389	2389	2389	2389	2389
Ligand	240	76	46	39	39	38	16	31	42	31
Solvent	322	399	328	330	341	394	383	302	350	197
R.m.s. deviations										
Bonds (Å)	0.010	0.006	0.007	0.006	0.005	0.005	0.009	0.010	0.006	0.014
Angles (°)	1.21	0.98	1.00	0.94	0.92	0.92	1.10	1.15	0.99	1.28
Average *B* factors (Å^2^)										
Protein	15.2	13.3	14.5	12.7	13.8	13.6	12.1	12.1	16.9	19.5
Ligand	18.1	32.5	29.6	35.2	33.0	39.6	17.8	35.0	49.9	144.4
Waters	28.8	29.8	32.9	30.3	29.3	30.1	28.0	25.4	35.7	27.6
Ramachandran plot (%)										
Outliers	0	0	0	0	0	0	0	0	0	0
Allowed	1	1	1	1	1	1	1	1	1	1
Favored	99	99	99	99	99	99	99	99	99	99
