# Supplementary material for: Frag4Lead: growing crystallographic fragment hits by catalog using fragment-guided template docking
Source: Acta Crystallogr D Struct Biol. 2021 Aug 23;77(Pt 9):1168–82. doi: 10.1107/S2059798321008196 (PMC8411975; doi:10.1107/S2059798321008196)
Supplement: Supplementary file 1 [file d-77-01168-sup1.pdf]

STRUCTURAL BIOLOGY

Volume 77 (2021)

Supporting information for article:

Frag4Lead: growing crystallographic fragment hits by catalog using fragment-guided template docking

Alexander Metz, Jan Wollenhaupt, Steffen Glöckner, Niki Messini, Simon Huber, Tatjana Barthel, Ahmed Mehrabed, Hans-Dieter Gerber, Andreas Heine, Gerhard Klebe and Manfred S. Weiss

Table S1. Acquired follow-up candidates, affinities, providers and purities.

Follow-Up Compound	*LE*[a]	K_d[b] [μM]	CAS Number	Salt	Provider	Provider Catalog ID	Purity[c] [%]
**FU$_5$-1** · HCl	0.39	6.4	40227-56-3	1xHCl	resynthesized	resynthesized	98.5[d]
**FU$_5$-1**	[e]	[e]	46886-52-6		Vitas-M	STL231607	90[f]
**FU$_5$-2**	0.37	970	3468-11-09		AK Scientific	P175	95
**FU$_5$-3**	0.39	740	14352-51-3		ENAMINE	EN300-43093	90
**FU$_5$-4**	0.31	400	879916-45-7		Vitas-M	STK594879	90[f]
**FU$_5$-5**			571161-36-9		Vitas-M	STK583580	90[f]
**FU$_{41}$-1**			919949-40-9		ENAMINE	Z106906918	90
**FU$_{41}$-2**			1007745-42-7		UORSY	PB106986608	n/a
**FU$_{41}$-3**			850020-65-4		ENAMINE	Z46204096	90
**FU$_{41}$-4**			1105566-03-7		ENAMINE	Z46442873	90[f]
**FU$_{41}$-5**			702651-79-4		ChemDiv	C094-0062	n/a[f]
**FU$_{41}$-6**			793678-62-3		ENAMINE	Z44502465	90
**FU$_{58}$-1**	0.17	450	1377929-97-9		ChemBridge	57162056	90
**FU$_{58}$-2**			1331991-20-8		ChemBridge	69271435	n/a
**FU$_{58}$-3**	(0.19) [g]	(1040)[g]	1244921-80-9		ChemBridge	58887845	90
**FU$_{58}$-4**			1360217-23-7		ChemBridge	22615646	90
**FU$_{58}$-5**			1332207-28-9		ChemBridge	55009691	n/a
**FU$_{58}$-6**			1378100-25-4		ChemBridge	17104240	n/a
**FU$_{58}$-7**			1269257-22-8		ChemBridge	33257899	n/a
**FU$_{58}$-8**			1005631-30-0		Vitas-M	STL398417	n/a[f]
**FU$_{58}$-9**			1355873-93-6		ENAMINE	Z1156178129	90
**FU$_{66}$-1**	0.21	160	1355830-29-3		ENAMINE	Z906038686	90
**FU$_{66}$-2**			2034580-57-7		Life Chemicals	F6482-6629	90
**FU$_{66}$-3**			2034475-57-3		Life Chemicals	F6482-6686	90
**FU$_{66}$-4**			2034307-19-0		Life Chemicals	F6482-6819	90
**FU$_{66}$-5**			1375192-22-5		ENAMINE	Z908233534	90
**FU$_{66}$-6**			1375215-10-3		ENAMINE	Z907996506	90
**FU$_{290}$-1**	0.44	7.2	10465-18-6	2xHCl	ChemBridge	5111431	90[f]
**FU$_{290}$-2**	0.40	160	1417634-38-8	1xHBr	Vitas-M	STL301596	n/a

[a] Ligand efficiency in kcal mol^{-1} $atom^{-1}$

[b] K_d value determined by ITC

[c] Purity reported by MolPort if not stated otherwise. “n/a”: no purity information available

[d] Determined by ^{1}H-qNMR

[e] Affinity only determined for resynthesized **FU$_5$-1** · HCl

[f] Identity verified by NMR provided by vendor

[g] Measurement is not reliable as crystallographic finding indicates only binding of impurity

Synthesis of compound FU_5-1

i.e. (Z)-3-amino-1-(2-phenylhydrazineylidene)-1*H*-isoindol-2-ium chloride

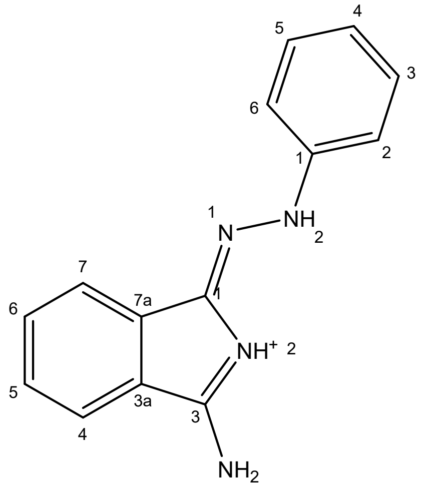

(*Z*)-3-amino-1-(2-phenylhydrazineylidene)-1 *H*-isoindol-2-ium
Chemical Formula: $C_{14}H_{13}N_4^+$
Exact Mass: 237.11
Molecular Weight: 237.29
m/z: 237.11 (100.0%), 238.12 (15.1%), 238.11 (1.5%), 239.12 (1.1%)
Elemental Analysis: C, 70.87; H, 5.52; N, 23.61

The target compound (**FU_5-1** · HCl) was prepared by a slightly modified literature procedure (Biitseva et al. 2013, Wolf, W. & Vollmann, H. 1956) as follows:

A stirred mixture of 1,3-diiminoisoindole (0.73 g, 5.0 mmol) and phenylhydrazine (0.54 g, 5.0 mmol) in MeOH (30 mL) was refluxed for 2 h. After cooling to RT, aqueous 1 M HCl (60 mL, 60 mmol) was added and the mixture stirred for 15 min at RT. The resulting yellow-orange precipitate was filtered through a glass filter (G3), thoroughly washed with water, then with a 1:1 mixture of water/MeOH and finally dried *in vacuo* over P_4O_{10} giving rise to 0.92 g (67.5 %) of a yellow-orange solid.

m.p. 228 °C (decomp.; (Biitseva *et al.*, 2013): 172 °C); at ca. 175 °C morphism into needles! ^{1}H NMR (DMSO-d_6, 500.1 MHz): δ = 13.30 (bs, 1H), 11.41 (bs, 1H), 10.61 and 10.36 (2 x bs, merged, 2H), 8.29 (d, $^3J_{H,H}$ = 7.8 Hz, 1H), 7.97 (d, $^3J_{H,H}$ = 7.6 Hz, 1H), 7.82 (t, $^3J_{H,H}$ = 7.8 Hz, 1H), 7.65 (t, $^3J_{H,H}$ = 7.6 Hz, 1H), 7.37-7.27 (m, 4 H), 6.95-6.87 (sm, 1H). ^{13}C NMR (DMSO-d_6, 100.5 MHz): δ = 158.6, 144.0, 134.4, 134.2, 131.2, 129.2, 129.1, 125.7, 124.2, 120.7, 120.5, 112.7. MS (ESI+) *m/z* (%): 237 (93) $[M]^+$. HRMS (ESI+): *m/z* calculated for $C_{14}H_{13}N_4^+$ 237.1135 $[M]^+$; found 237.1139. Elemental analysis calculated (%) for $C_{14}H_{13}N_4Cl$ (272.74): C 61.65, H 4.80, N 20.54, Cl, 13.00; found: C, 61.13; H, 5.04; N, 20.27; Cl, 12.86

Substance assay, determined by ^{1}H qNMR (DMSO-d_6, 500.1 MHz, reference standard maleic acid TraceCert®): 98.5 %

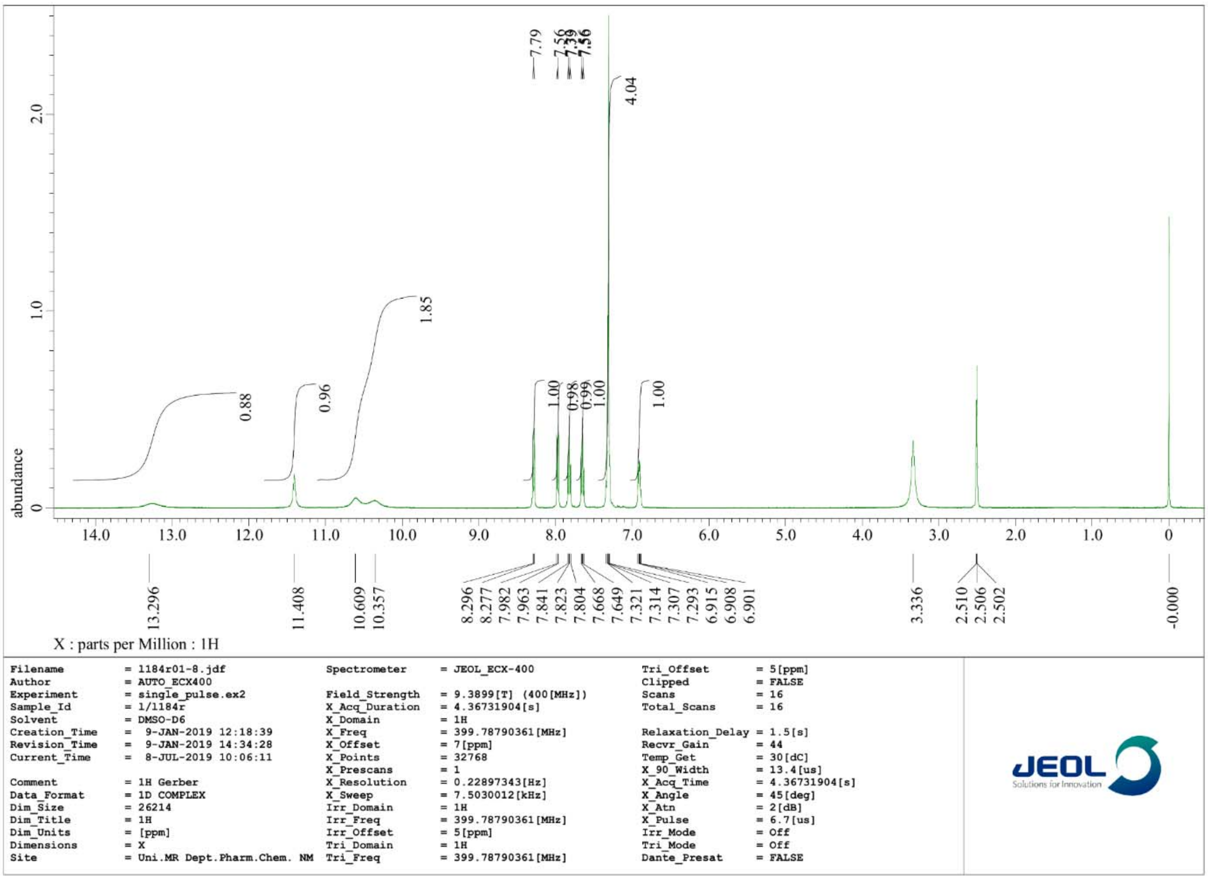

Figure S1. ^{1}H NMR spectrum of **FU$_5$-1** · HCl.

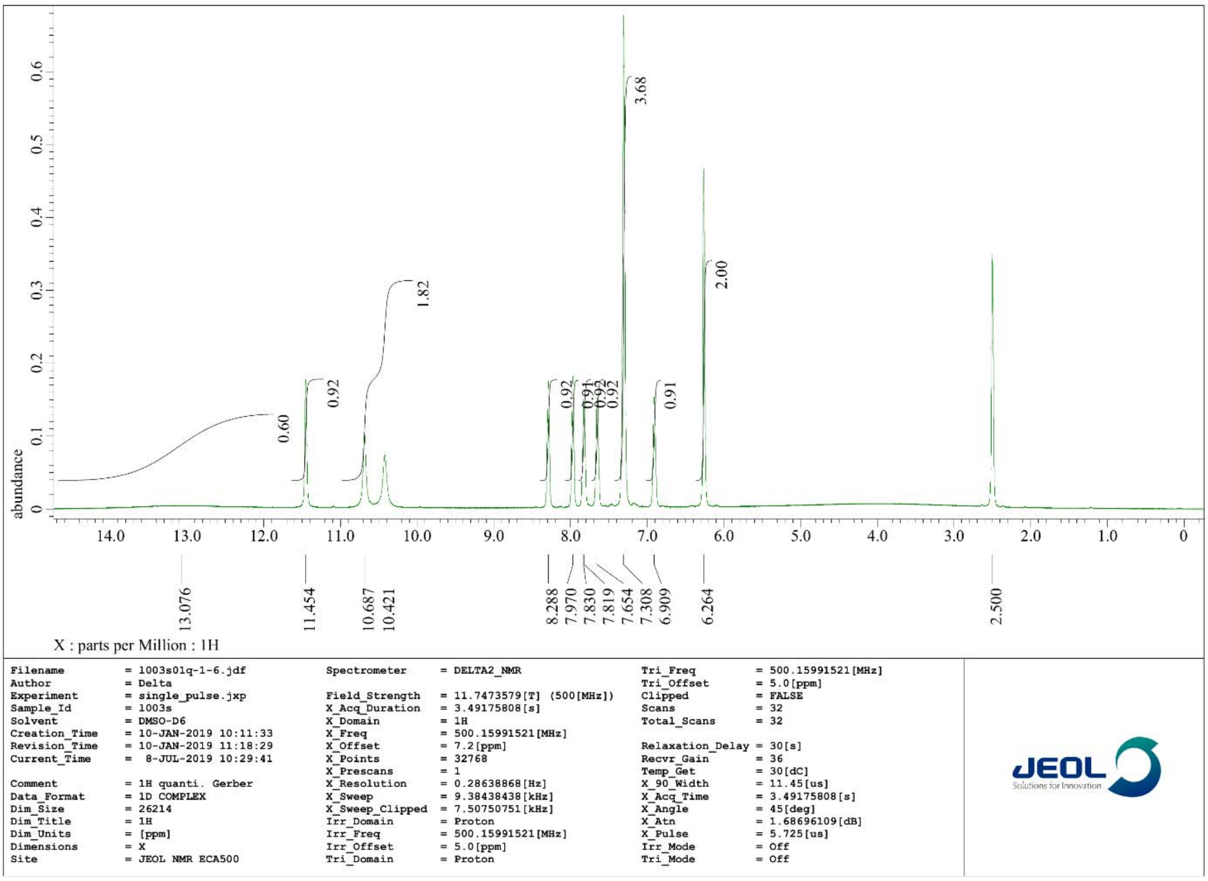

Figure S2. ^{1}H qNMR spectrum of **FU$_5$-1** · HCl.

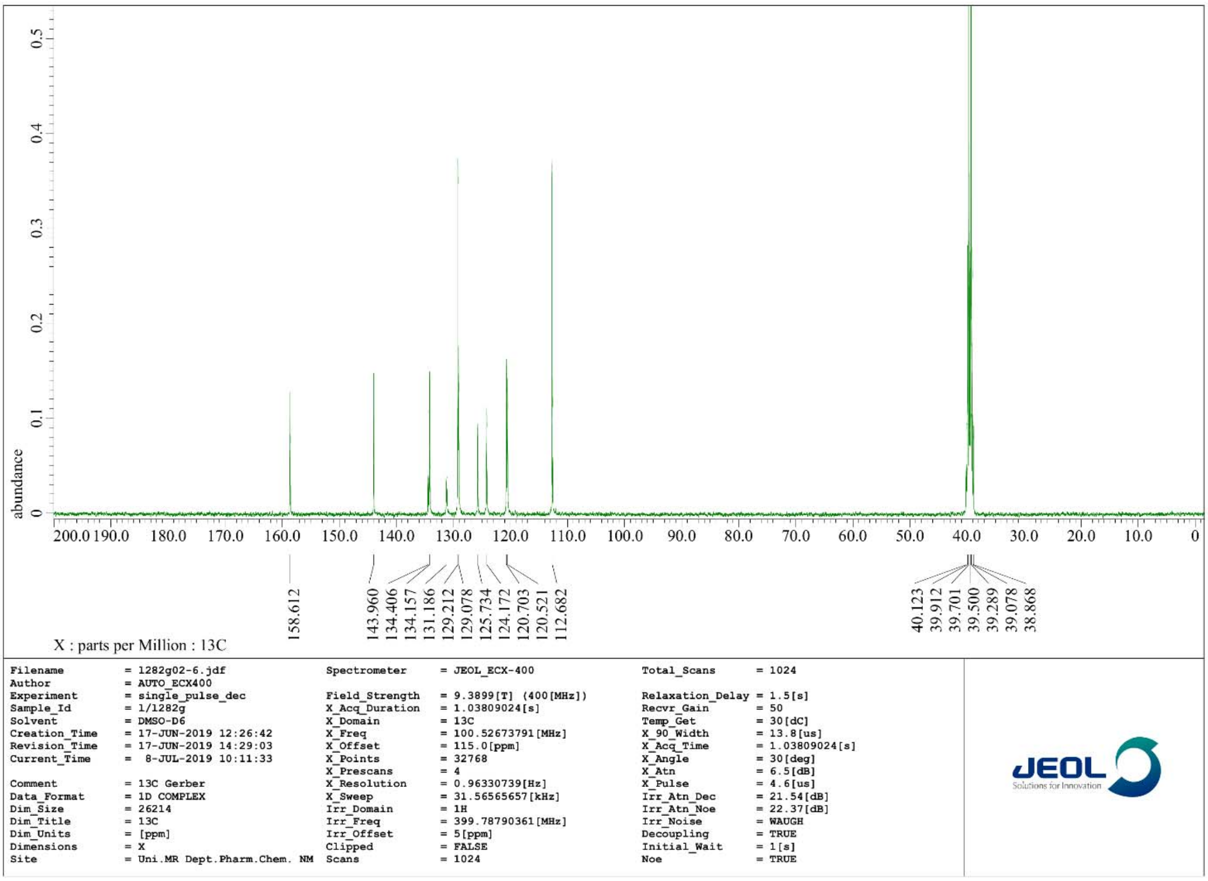

Figure S3. ^{13}C NMR spectrum of **FU$_5$-1** · HCl.

Table S2. Details of ITC measurements.

Follow-Up Compound	Titration Type	Protein Concentration in Cell	Follow-Up Ligand Concentration in Cell	Ligand Concentration in Syringe	Final Stoichiometry (Ligand:Protein)
**FU$_5$-1** · HCl	direct	25 µM[a]	–	500 µM[a]	4
**FU$_5$-2**	displacement	50 µM	2000 µM	500 µM	2
**FU$_5$-3**	displacement	50 µM	2000 µM	500 µM	2
**FU$_5$-4**	direct	50 µM	–	2000 µM	8
**FU$_{58}$-1**	displacement	50 µM	2000 µM	500 µM	2
**FU$_{58}$-2**[b]	–	–	–	–	–
**FU$_{58}$-3**	displacement	50 µM	2000 µM	500 µM	2
**FU$_{66}$-1**	displacement	50 µM	2000 µM	500 µM	2
**FU$_{290}$-1**	direct	50 µM	–	1000 µM	4
**FU$_{290}$-2**	displacement	50 µM	2000 µM	500 µM	2

[a] 0.1% (v/v) Tween 20 in syringe and cell.

[b] Not enough substance available for ITC.

Table S3. Fitting statistics for ITC titration curves

Compound	Global reduced chi-square[a]	Weighted local rmsd[a]	Stochiometry[b] (B:A)	n[c]	K_d [µM] (SD)[d]
**FU_5-1**[e]	0.026	28.6	0.875	25	6.4 (± 0.06)
**FU_5-1**[e]	0.024	38.7	0.915	25	6.4 (± 0.06)
**FU_5-1**[e]	0.003	37.7	0.858	25	6.4 (± 0.06)
**FU_5-2**	0.228	135.3	0.991	25	970 (± 79)
**FU_5-2**	0.357	268.2	0.972	25	970 (± 79)
**FU_5-2**	0.284	238.2	1.018	25	970 (± 79)
**FU_5-3**	0.107	240.5	1.052	13	740
**FU_5-4**	0.014	18.6	1.000[g]	38	400
**FU_{58}-1**	0.134	197.5	1.081	24	450
**FU_{58}-3**	0.498	396.7	1.068	24	1040
**FU_{66}-1**	0.052	226.0	1.142	24	160
**FU_{290}-1**	0.075	186.9	0.816	74	7.2
**FU_{290}-2**	0.065	41.4	0.871	25	160
Reference (SAP114)	0.346	193.2	1.001	24	680

Isotherm fitting statistics of ITC data, analyzed via SEDPHAT 10.58d (Houtman *et al.*, 2007).
[a] Goodness of fit parameters (global reduced chi-square and weighted local rmsd) determined with SEDPHAT 10.58d are comparable to those listed for the established reference compound SAP114.
[b] Stochiometry of the ligand protein A and the ligand B at the inflection point of the sigmoidal fitting curve
[c] Number of used data points corresponding to injections of the ITC experiment
[d] K_d values. Standard deviation (SD) is only listed for **FU_5-1** and **FU_5-2**, which were measured as triplicates.
[e] For **FU_5-1**, global reduced chi-square and weighted local rmsd are listed for comparison only and the listed K_d value was determined by global fit with the AFFINImeter suite (version 2.1710; Muñoz & Piñeiro, 2018).
[f] For **FU_5-4** the stoichiometry was fixed to a value of 1 to achieve a satisfactory fit

ΔH and $T\Delta S$ values are not reported for this experiment following the reasoning of Krimmer and Klebe (2015).

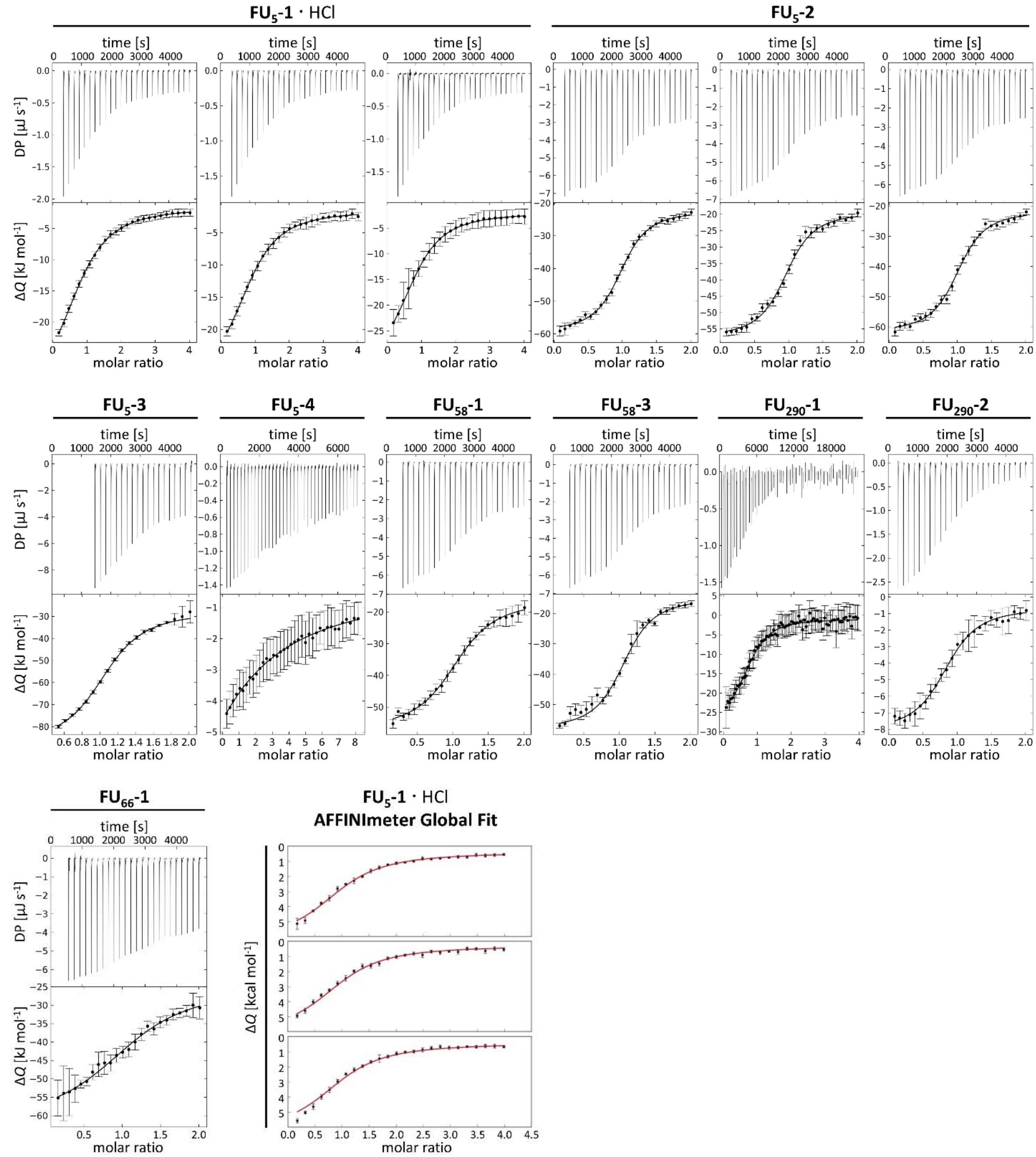

Figure S4. ITC thermograms for crystallographically identified follow-up ligands. In all cases, the heat signals DP (in µJ s^{-1}) as a response by the release of the ligand into the protein solution is shown over the course of the experiment along with the integrated heat signals of the injections *ΔQ* (in kJ mol^{-1}). **FU_5-1** · HCl and **FU_5-2** were measured in triplicate, all other ligands as single measurements (*n* = 1), in due regard of the limited resilience of the results of the latter. The noisy baseline of the ITC experiments for **FU_{290}-1** was allegedly due to its low purity (> 90% according to provider) and could not be further improved.

Mass Spectrometry Analysis of $\mathbf{FU_{58}}$-3

Clear difference electron density and steric constraints in the crystal structure obtained by soaking **$\mathbf{FU_{58}}$-3** showed that **$\mathbf{FU_{58}}$-3b** had bound instead. The presence of **$\mathbf{FU_{58}}$-3b** in the commercially obtained sample of **$\mathbf{FU_{58}}$-3** (> 90% according to provider (ChemBridge, USA)) was demonstrated by mass spectrometry (MS) experiments (Figure S6) using a Q-Trap 2000 (Applied Biosystems, USA).

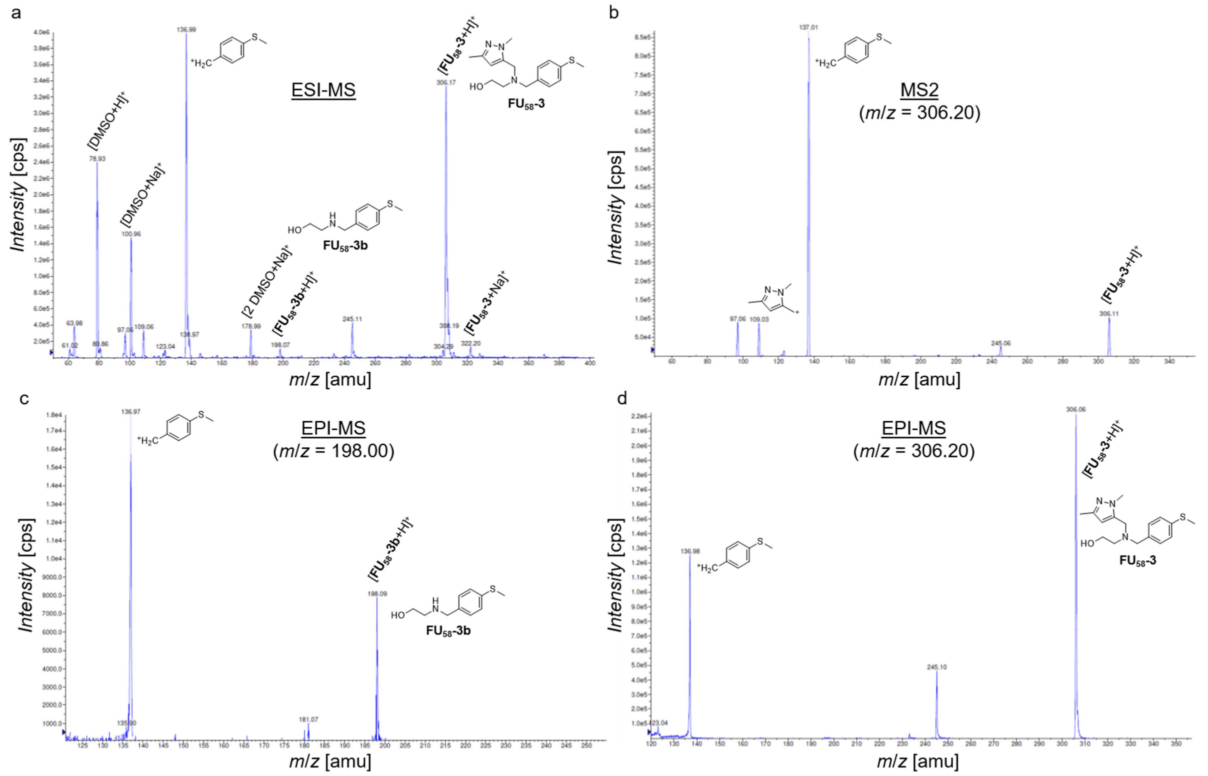

Figure S5. Mass spectrometry results of commercially obtained sample of **$\mathbf{FU_{58}}$-3**. a) ESI-MS. b) MS2. c) EPI-MS targeting *m*/*z* = 198.00. c) EPI-MS targeting *m*/*z* = 306.20.

In detail, **$\mathbf{FU_{58}}$-3** was dissolved in methanol from 1 M DMSO stock and characterized by electrospray ionization (ESI) MS (Figure S6a). This demonstrated the presence of **$\mathbf{FU_{58}}$-3** (calcd. for $C_{16}H_{24}N_3OS$ $[M\text{+H}]^+$ 306.16, found m/z = 306.17) and its substructure **$\mathbf{FU_{58}}$-3b** (2-((4-(methylthio)benzyl)amino)ethan-1-ol) (calcd. for $C_{10}H_{16}NOS$ $[M\text{+H}]^+$ 198.09, found m/z = 198.07). In addition, MS2 targeting **$\mathbf{FU_{58}}$-3** (m/z = 306.20; Figure S6b) demonstrated its presence (m/z = 306.11) as well as the presence of its fragmentation products (4-(methylthio)phenyl)methylium (calcd. for C_8H_9S $[M]^+$ 137.04, found m/z = 137.01) and (1,3-dimethyl-1H-pyrazol-5-yl)methylium (calcd. for $C_6H_9N_2$ $[M]^+$ 109.08, found m/z = 109.03). However, **$\mathbf{FU_{58}}$-3b** was not observed in this MS2 experiment. Finally, **$\mathbf{FU_{58}}$-3b** was clearly observed in enhanced product ion (EPI) MS targeting m/z = 306.20 (Figure S6c) but not in the EPI MS targeting m/z = 198.00 (Figure S6d). This indicates **$\mathbf{FU_{58}}$-3b** is not a decomposition fragment of **$\mathbf{FU_{58}}$-3** but instead an impurity of the obtained substance. As a side note, 4-(methylthio)benzylamine (calcd. for $C_8H_{12}NS$ $[M\text{+H}]^+$ 154.07), which could partially have matched the observed electron density, was not observed in any of the MS experiments. Thus, only **$\mathbf{FU_{58}}$-3b** was unambiguously attributed to the electron density and built into the crystal structure obtained by soaking **$\mathbf{FU_{58}}$-3** (PDB ID: 5SAQ).

Table S4. Retrospective, unbiased docking of follow-up compounds.

Starting Fragment	Follow-Up	fragment core at similar position as starting fragment?	# of FlexX poses	successfully HYDE-scored poses	HYDE-scored poses without bad clashes/ torsions	RMSD to crystallographic pose < 2 Å		
						total	in top 10 scored	in top3 scored
**F005**	**FU_5-1**	yes	500	405	403	59	0	0
	**FU_5-2**	yes	500	450	442	68	0	0
	**FU_5-3**	yes	467	413	412	71	0	0
	**FU_5-4**	no	448	381	378	0	0	0
**F058**	**FU_{58}-1**	no	235	125	113	0	0	0
	**FU_{58}-2**	no	403	319	287	0	0	0
**F066**	**FU_{66}-1**	no	333	202	127	0	0	0
**F290**	**FU_{290}-1**	yes	386	343	236	0	0	0
	**FU_{290}-2**	yes	500	440	398	25	3	0

Supplementary references

Biitseva, A., Groth, U. & Hordiyenko, O. (2013). *J. Heterocyclic Chem.* **50,** 1140-1145

Wolf, W. & Vollmann, H. (1956). *German Patent* DE 941845

Krimmer, S. G. & Klebe, G. (2015). *J. Comput. Mol. Des.* **29**, 867–883.
